# Supplementary figures and images for: Rapid and convenient detection of SARS-CoV-2 using a colorimetric triple-target reverse transcription loop-mediated isothermal amplification method
Source: PeerJ. 2022 Oct 10;10:e14121. doi: 10.7717/peerj.14121 (PMC9558625; doi:10.7717/peerj.14121)

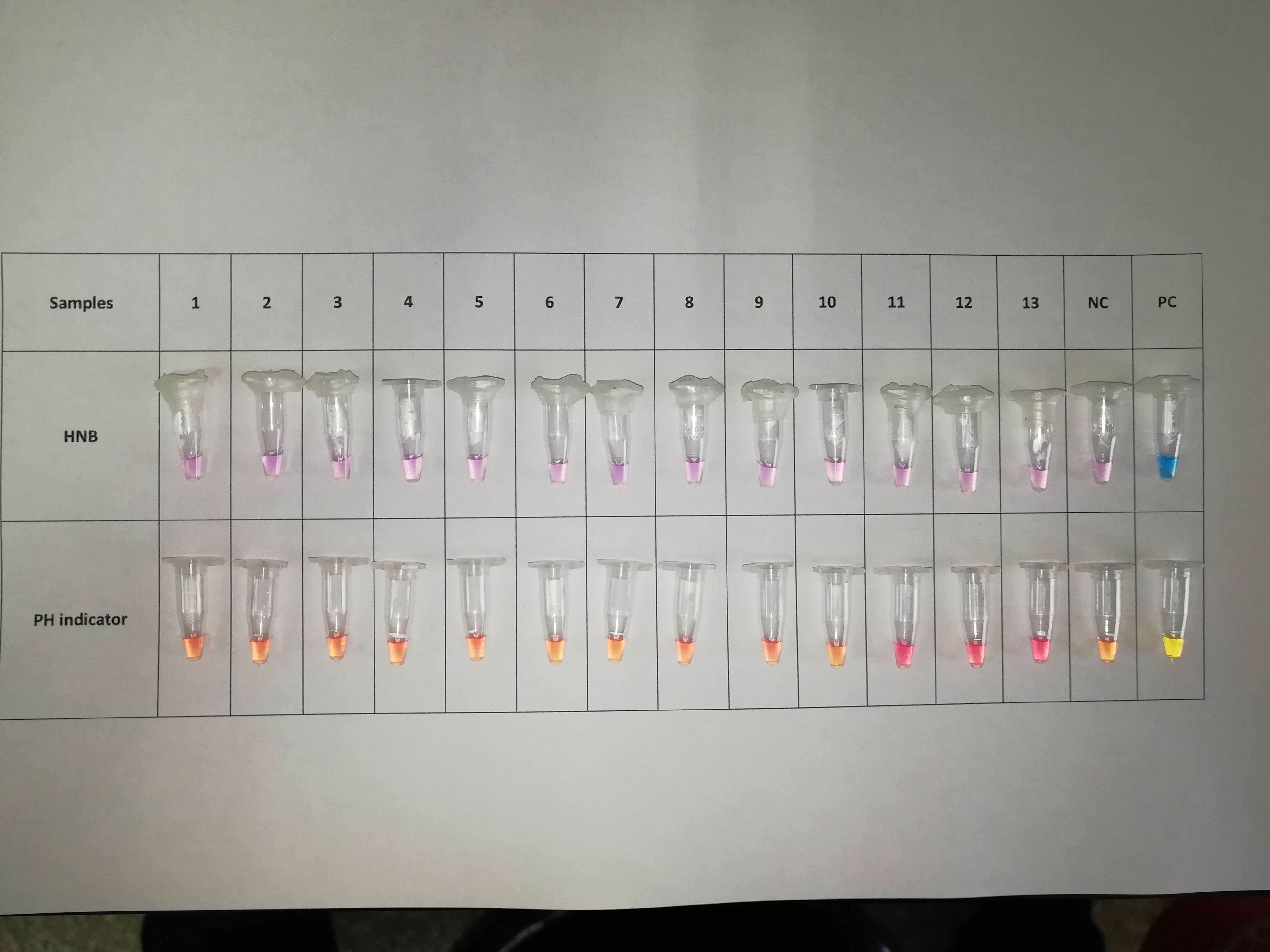

Supplement: Supplemental Information 1 — LC96P files can be opened and analyzed with LightCycler® 96 SW 1.1 software (roche.com). [file peerj-10-14121-s001.zip › Raw data/20210621_Specificity test of colorimetric triplex RT-LAMP assay.jpg]

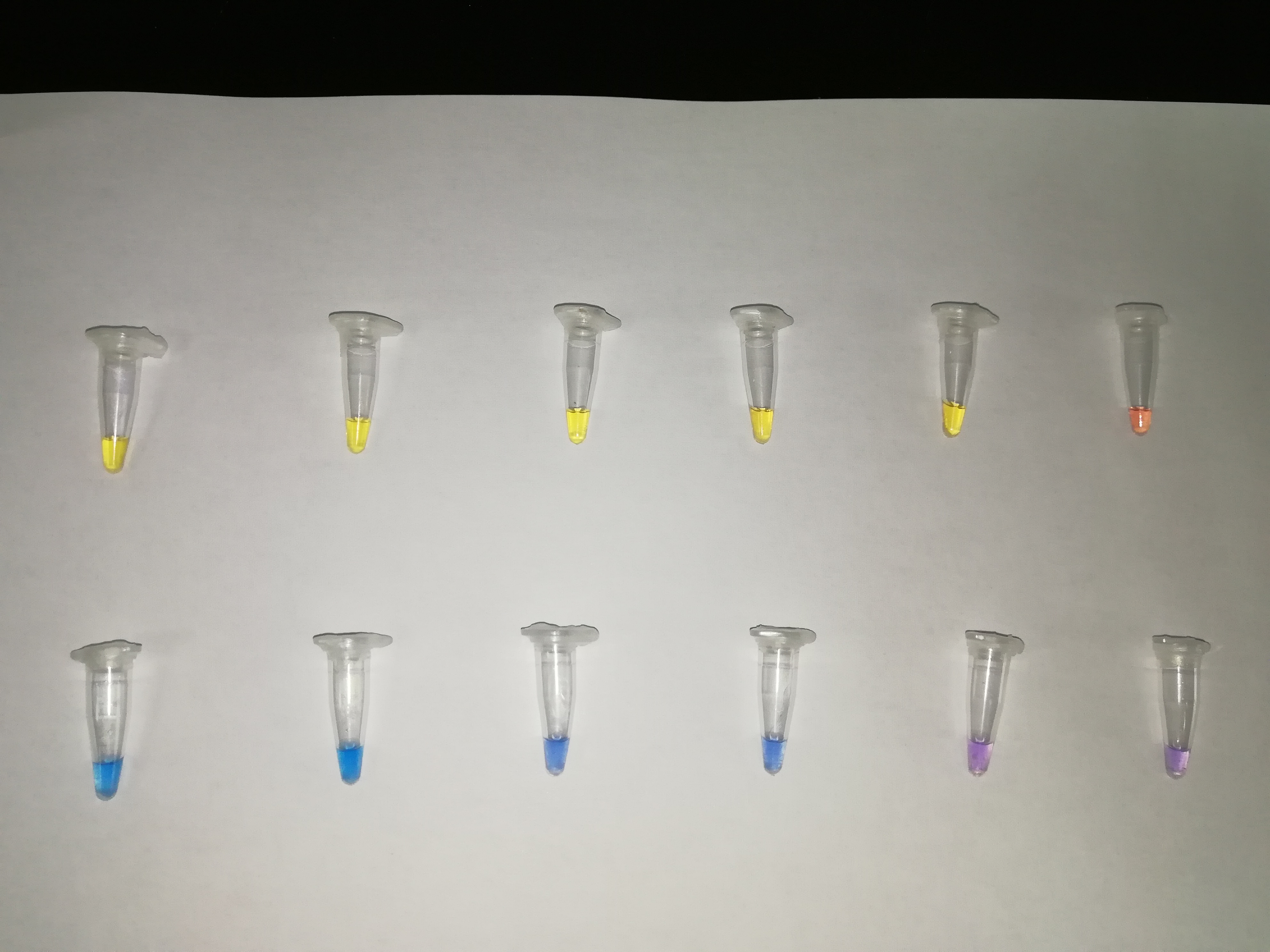

Supplement: Supplemental Information 1 — LC96P files can be opened and analyzed with LightCycler® 96 SW 1.1 software (roche.com). [file peerj-10-14121-s001.zip › Raw data/20210603-sensitivity test of colorimetric triplex RT-LAMP assay.jpg]

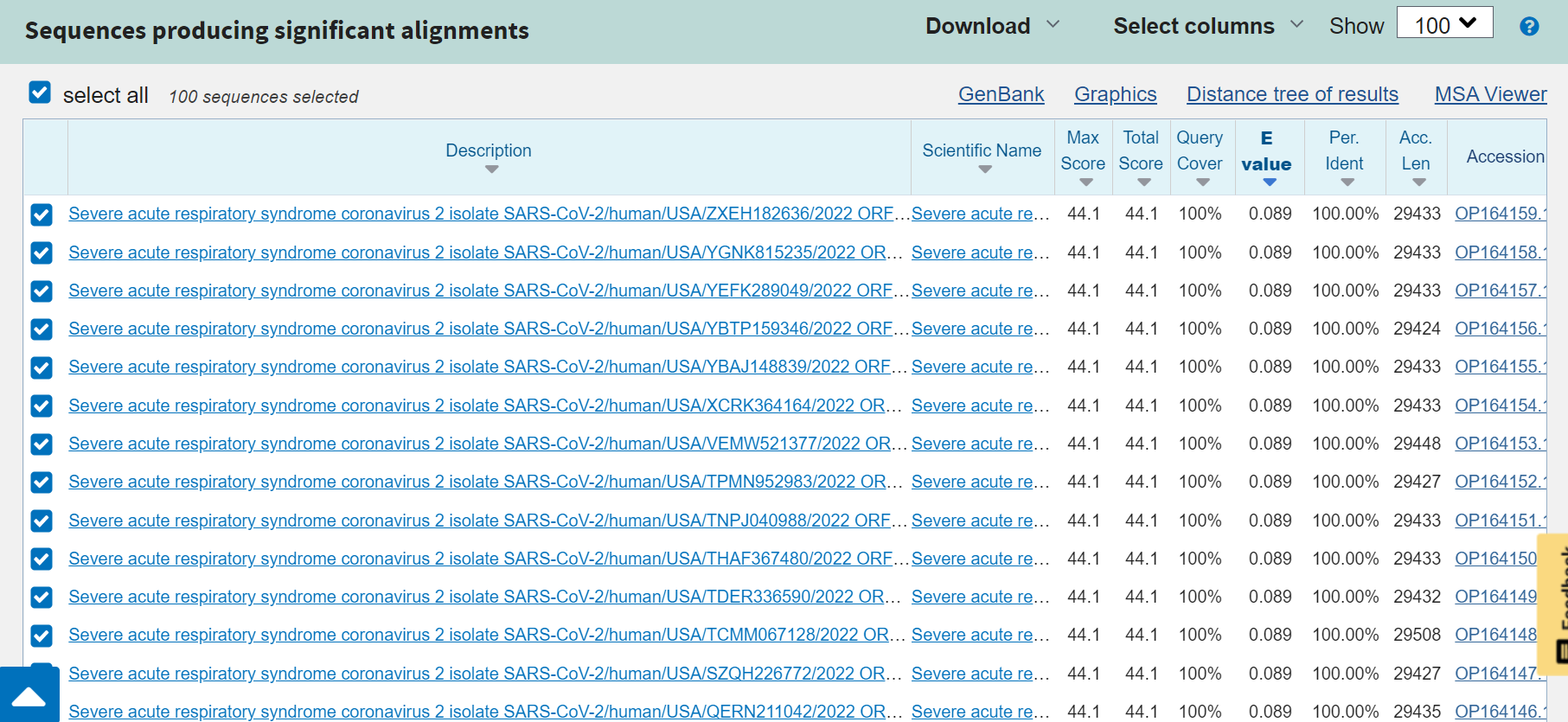

Supplement: Supplemental Information 2 [file peerj-10-14121-s002.zip › Supplemental 1-Blasting results of the LAMP primer sets/Set-1/B3 primer.png]

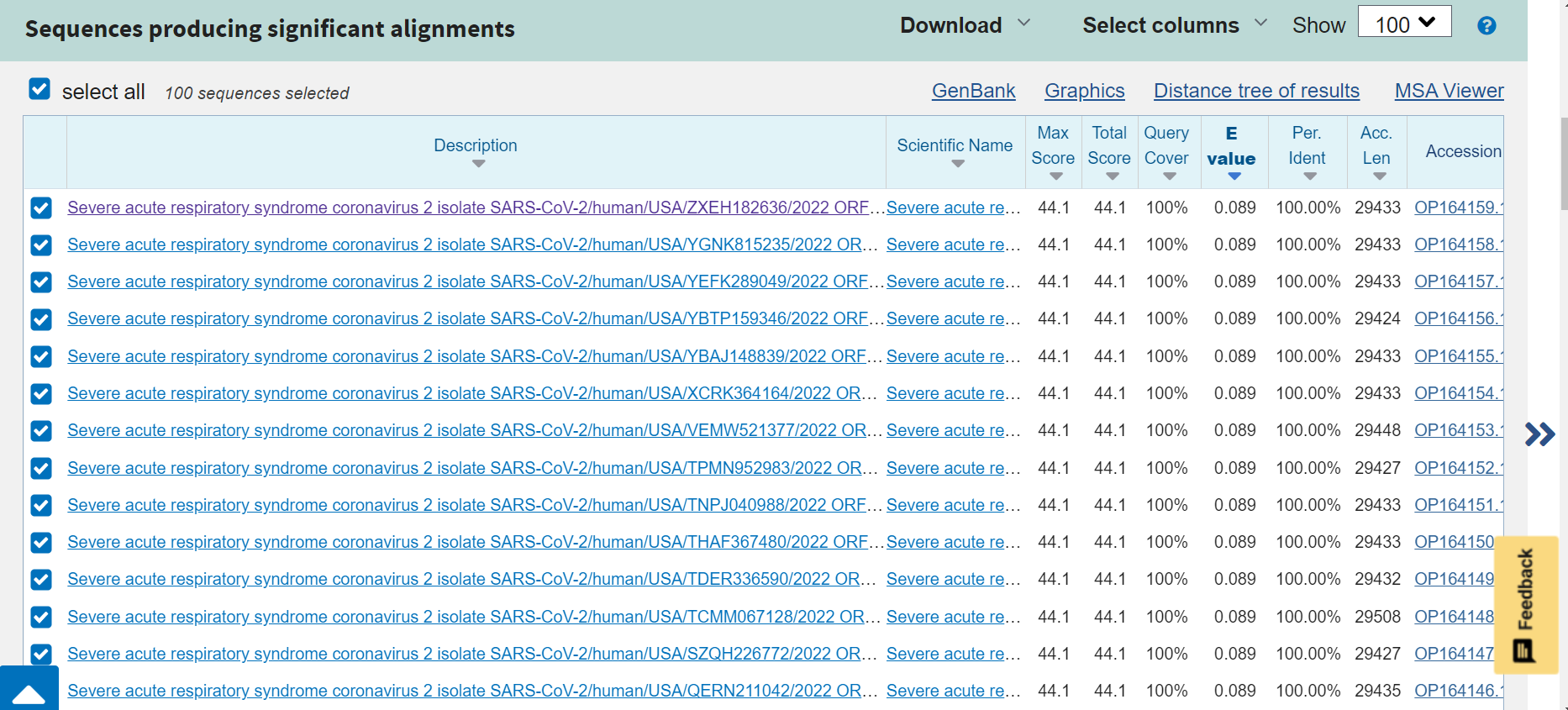

Supplement: Supplemental Information 2 [file peerj-10-14121-s002.zip › Supplemental 1-Blasting results of the LAMP primer sets/Set-1/BIP primer-B1c.png]

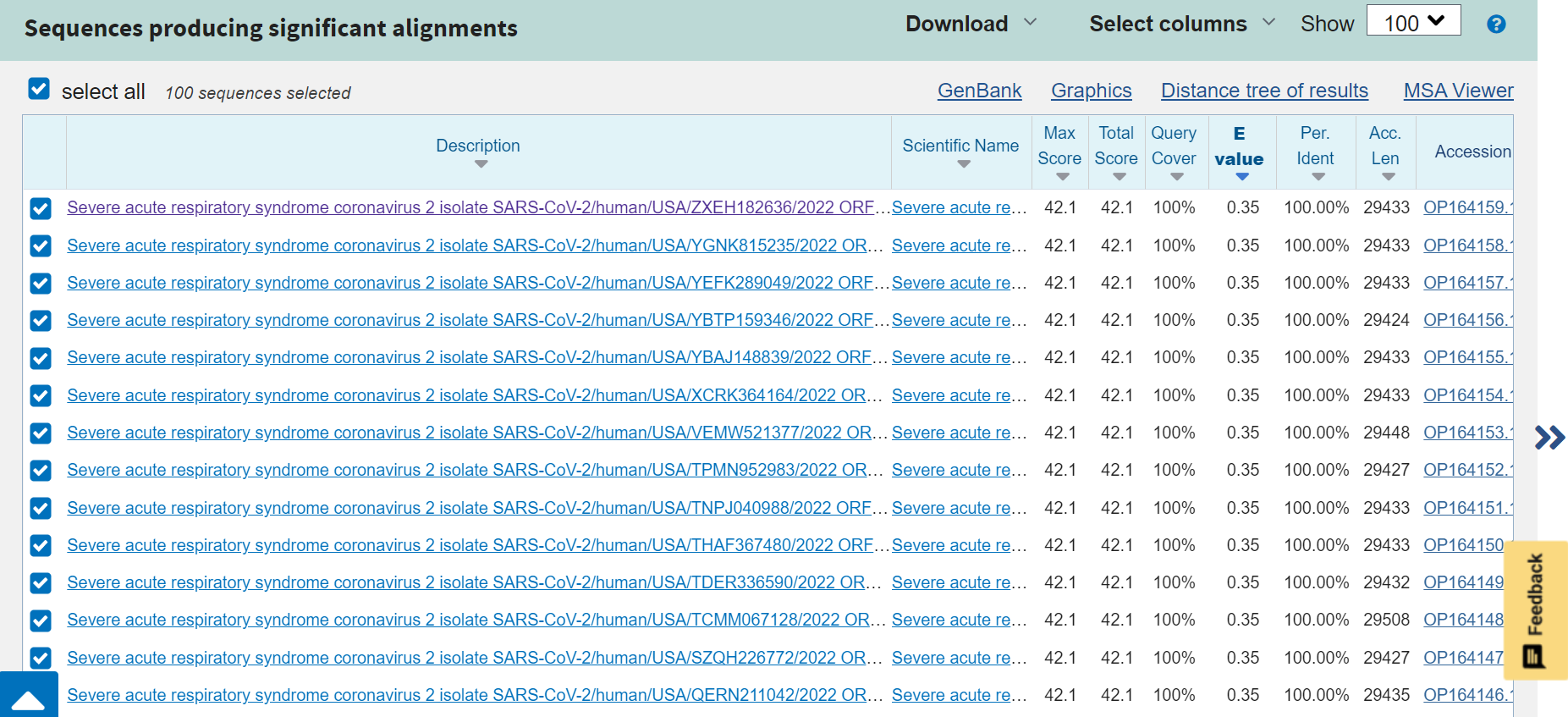

Supplement: Supplemental Information 2 [file peerj-10-14121-s002.zip › Supplemental 1-Blasting results of the LAMP primer sets/Set-1/BIP primer-B2.png]

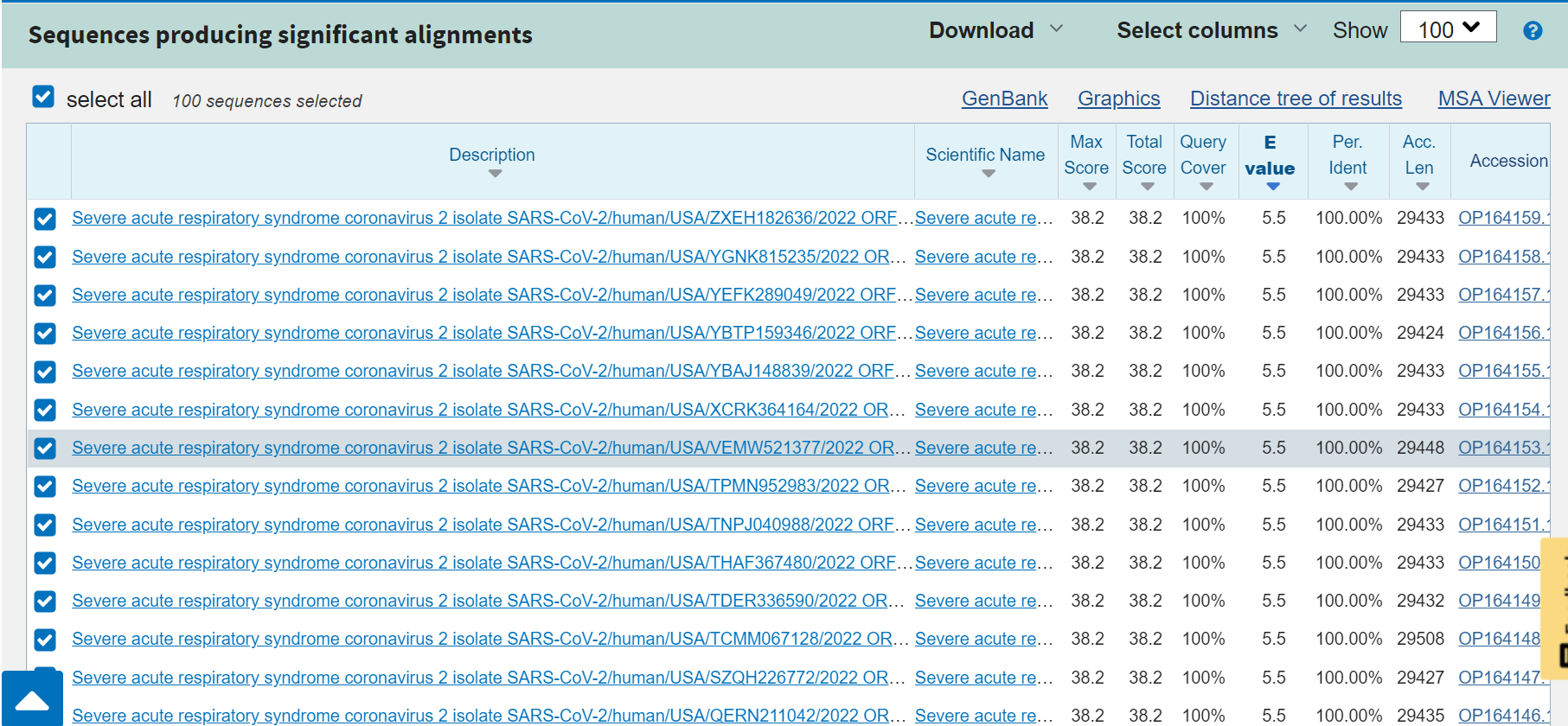

Supplement: Supplemental Information 2 [file peerj-10-14121-s002.zip › Supplemental 1-Blasting results of the LAMP primer sets/Set-1/F3 primer.png]

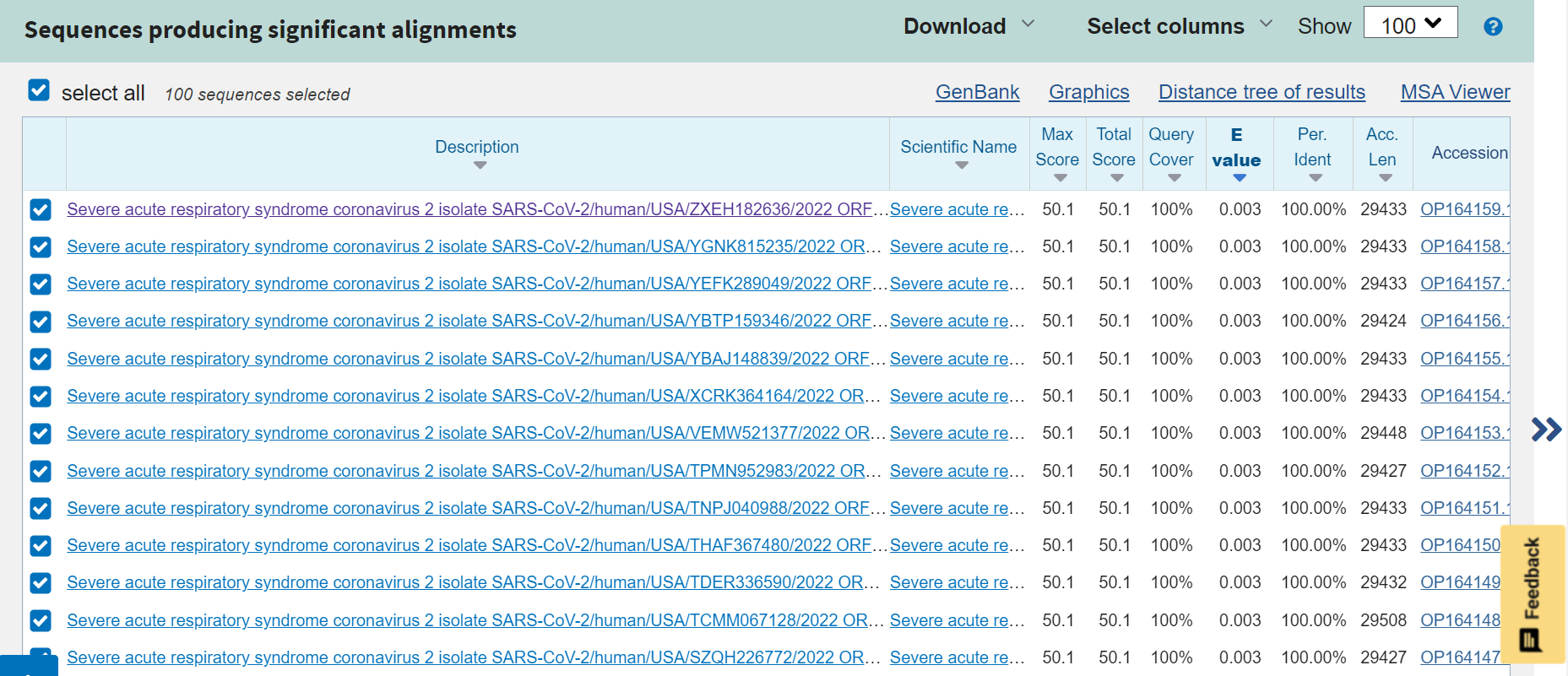

Supplement: Supplemental Information 2 [file peerj-10-14121-s002.zip › Supplemental 1-Blasting results of the LAMP primer sets/Set-1/FIP-primer-F1c.png]

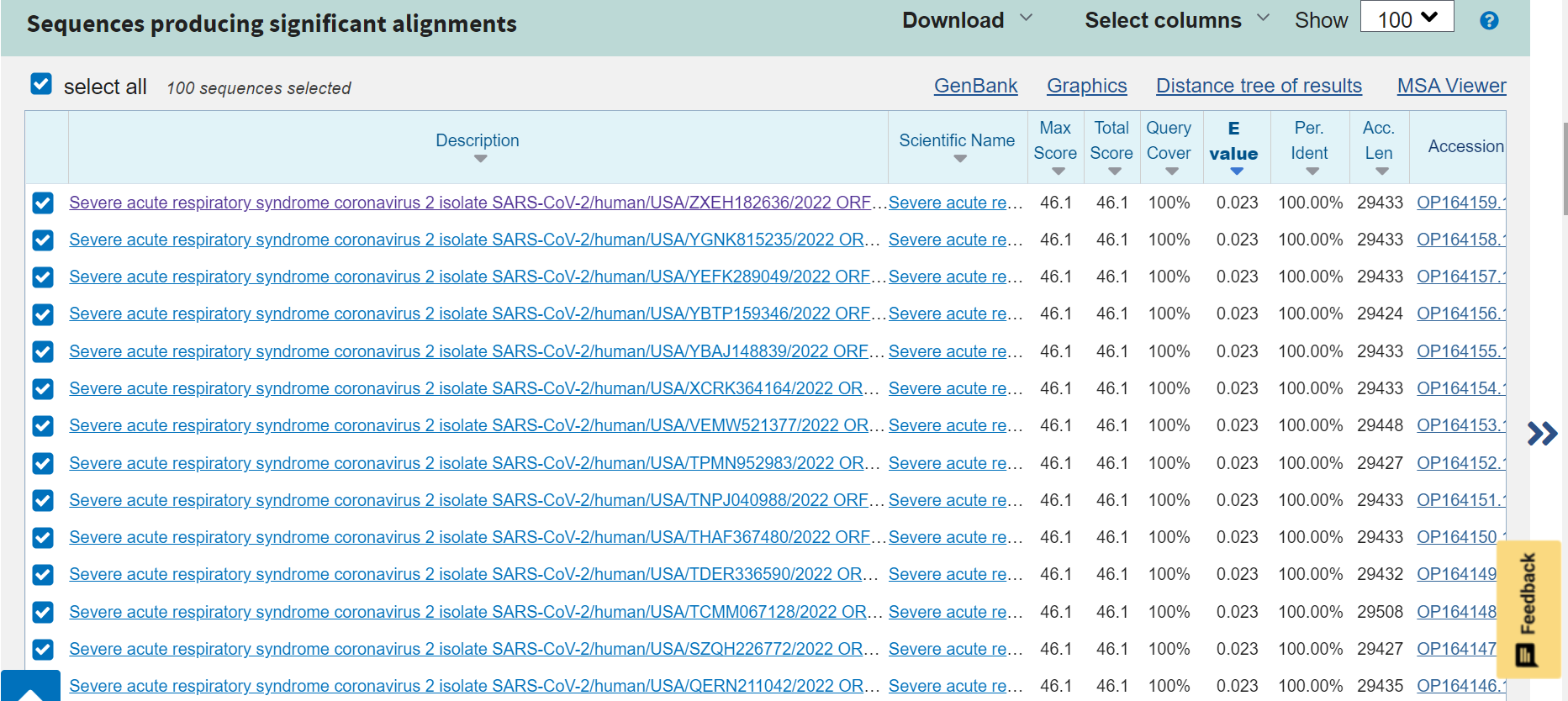

Supplement: Supplemental Information 2 [file peerj-10-14121-s002.zip › Supplemental 1-Blasting results of the LAMP primer sets/Set-1/FIP-primer-F2.png]

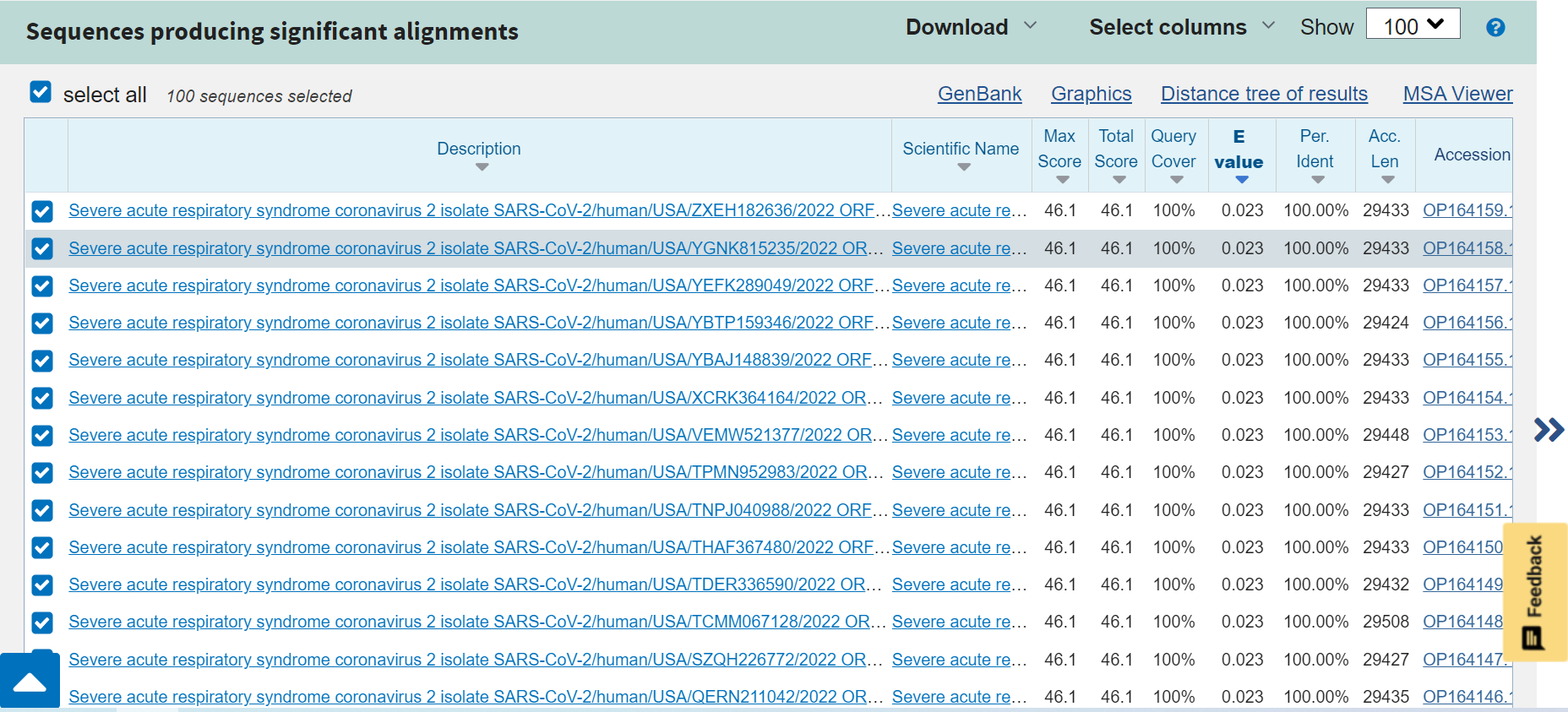

Supplement: Supplemental Information 2 [file peerj-10-14121-s002.zip › Supplemental 1-Blasting results of the LAMP primer sets/Set-1/LB primer.png]

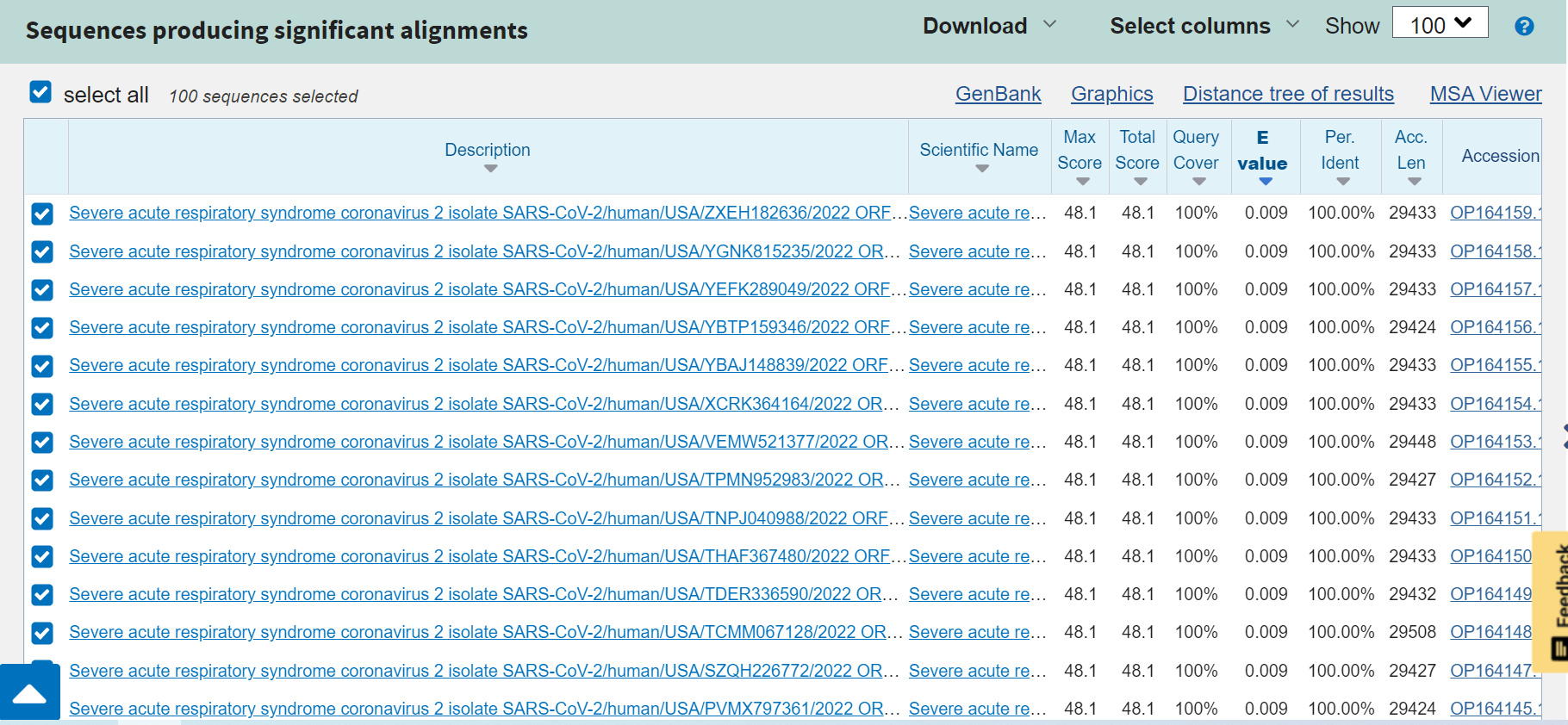

Supplement: Supplemental Information 2 [file peerj-10-14121-s002.zip › Supplemental 1-Blasting results of the LAMP primer sets/Set-1/LF primer.png]

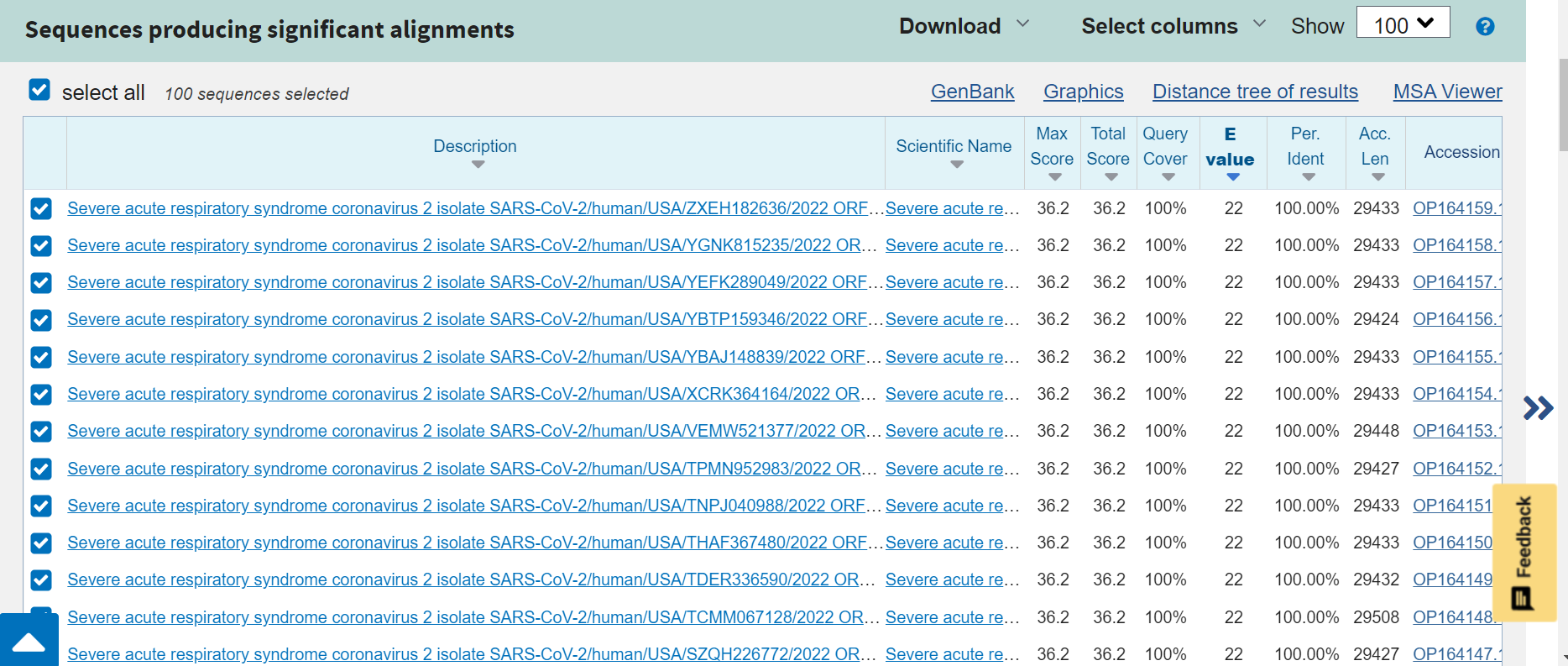

Supplement: Supplemental Information 2 [file peerj-10-14121-s002.zip › Supplemental 1-Blasting results of the LAMP primer sets/Set-2/B3 primer.png]

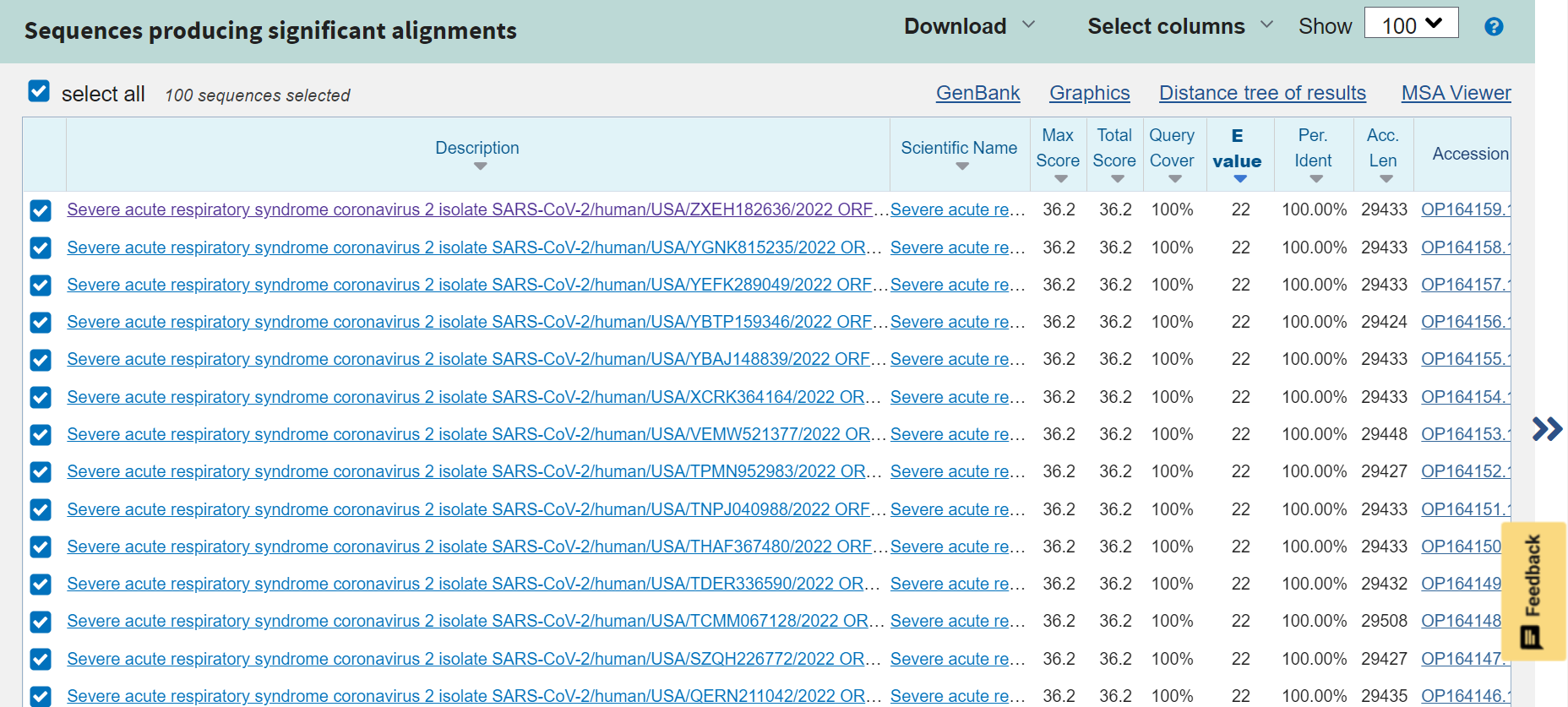

Supplement: Supplemental Information 2 [file peerj-10-14121-s002.zip › Supplemental 1-Blasting results of the LAMP primer sets/Set-2/BIP primer-B1c.png]

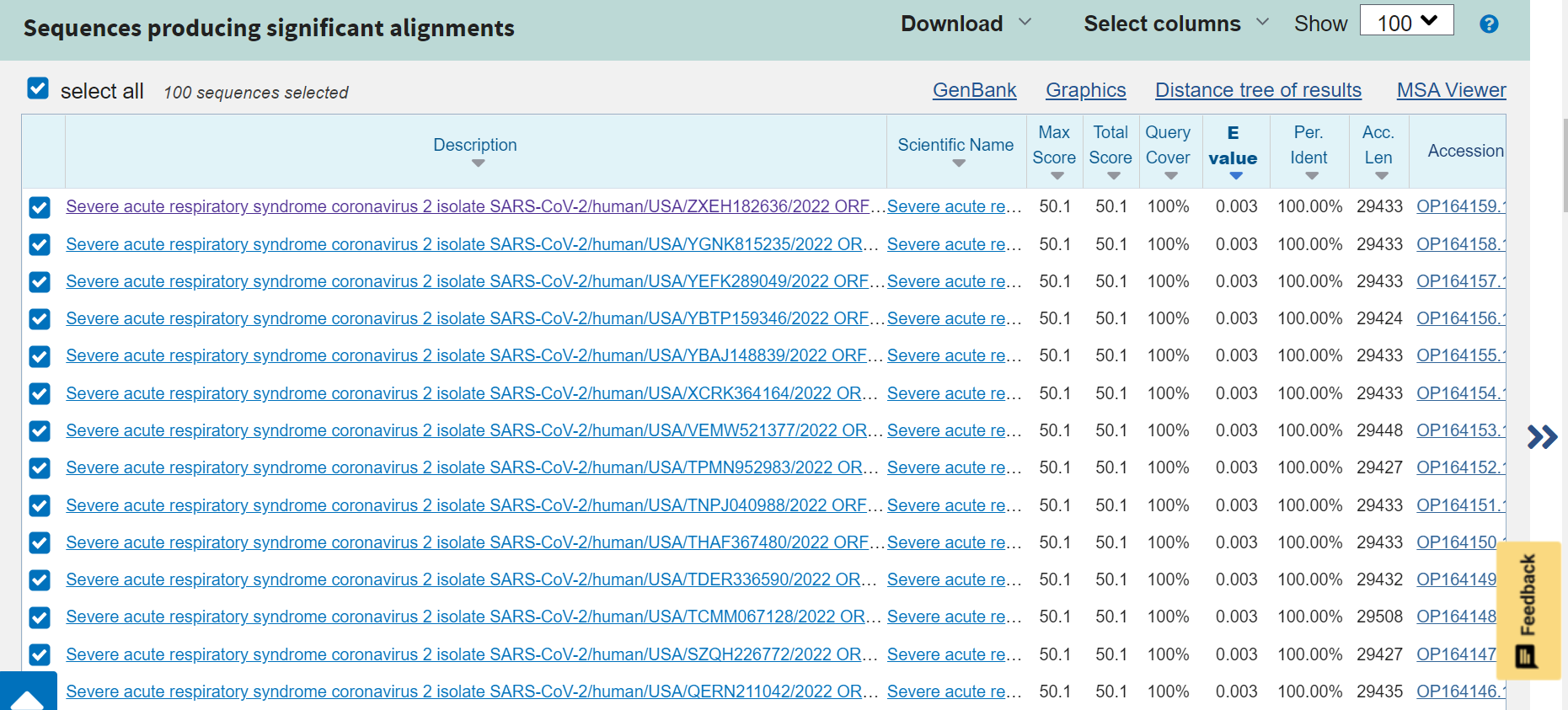

Supplement: Supplemental Information 2 [file peerj-10-14121-s002.zip › Supplemental 1-Blasting results of the LAMP primer sets/Set-2/BIP primer-B2.png]

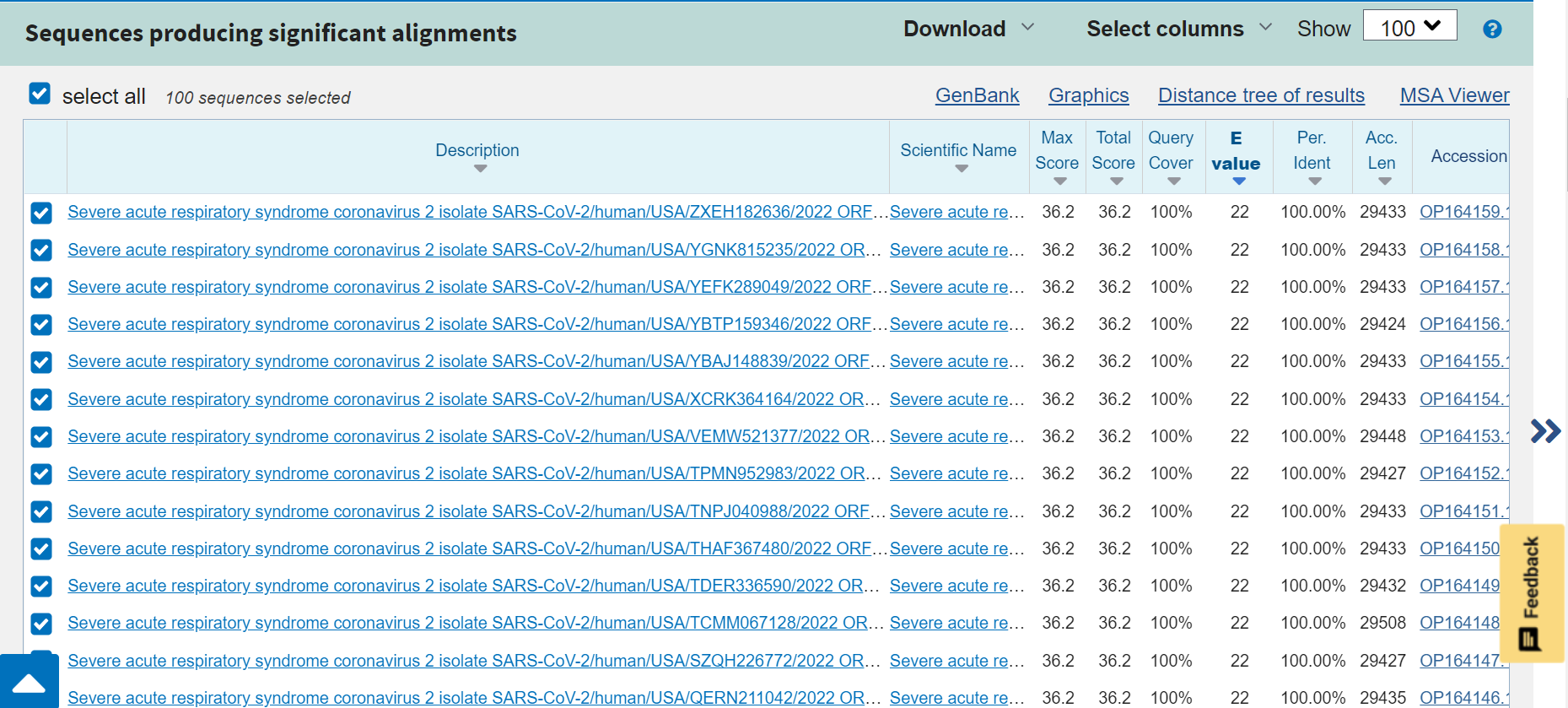

Supplement: Supplemental Information 2 [file peerj-10-14121-s002.zip › Supplemental 1-Blasting results of the LAMP primer sets/Set-2/F3 primer.png]

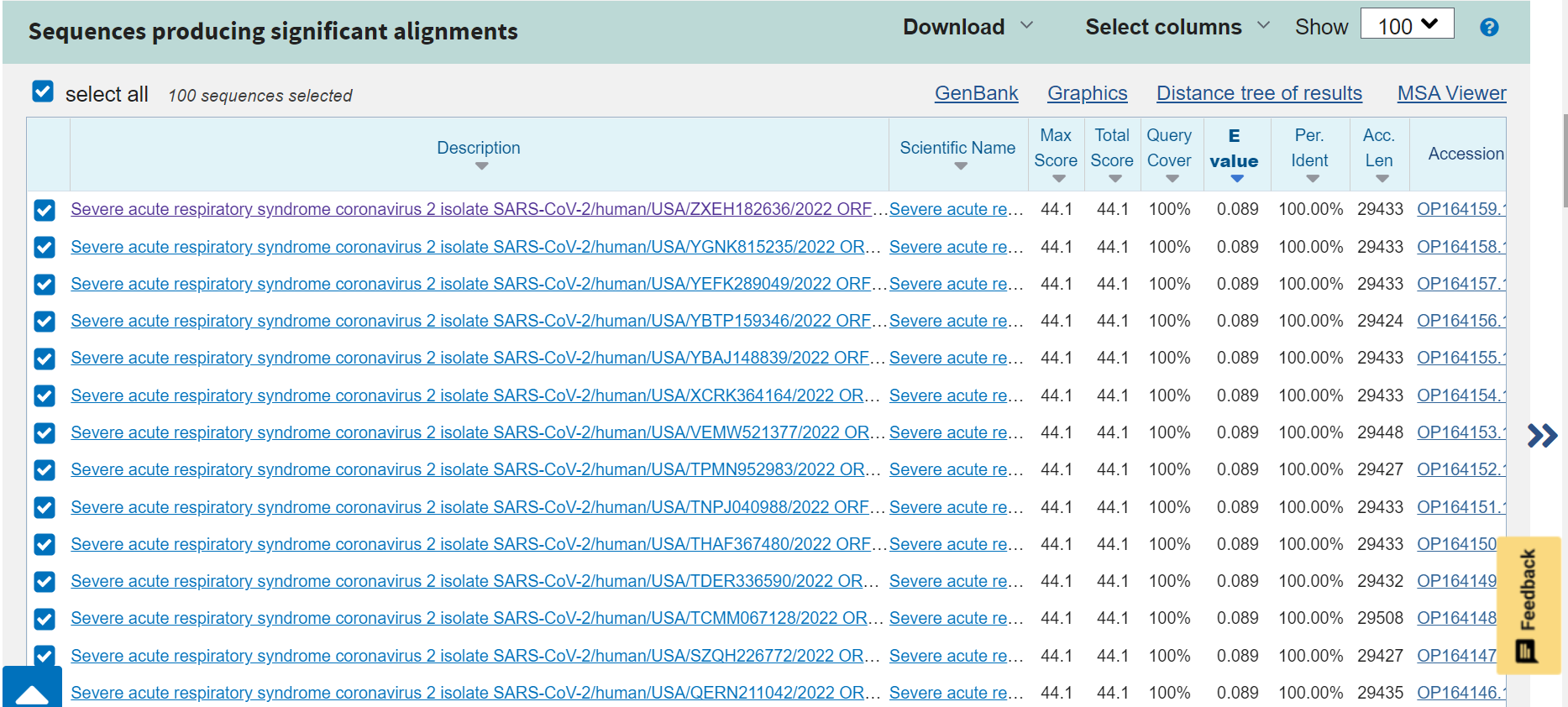

Supplement: Supplemental Information 2 [file peerj-10-14121-s002.zip › Supplemental 1-Blasting results of the LAMP primer sets/Set-2/FIP primer-F1c.png]

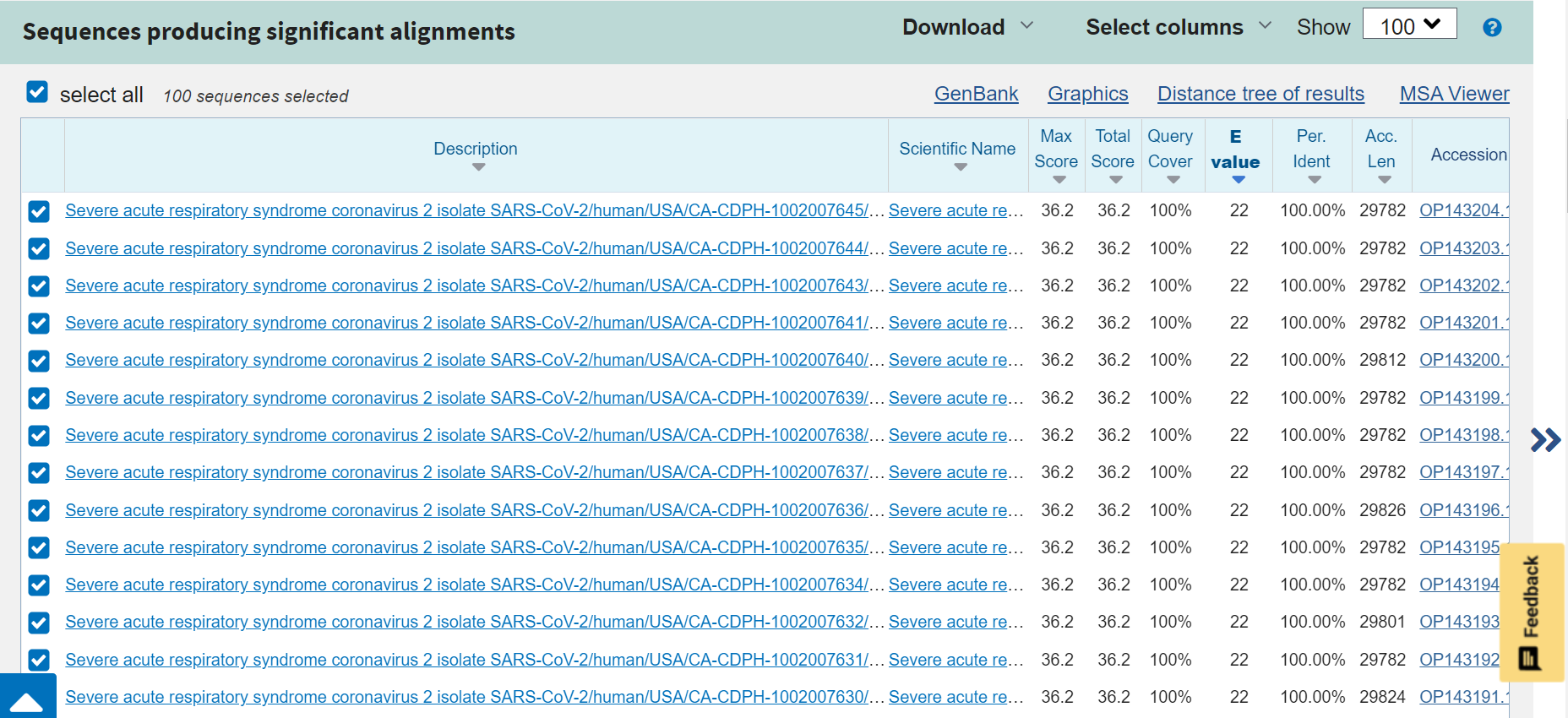

Supplement: Supplemental Information 2 [file peerj-10-14121-s002.zip › Supplemental 1-Blasting results of the LAMP primer sets/Set-2/FIP primer-F2.png]

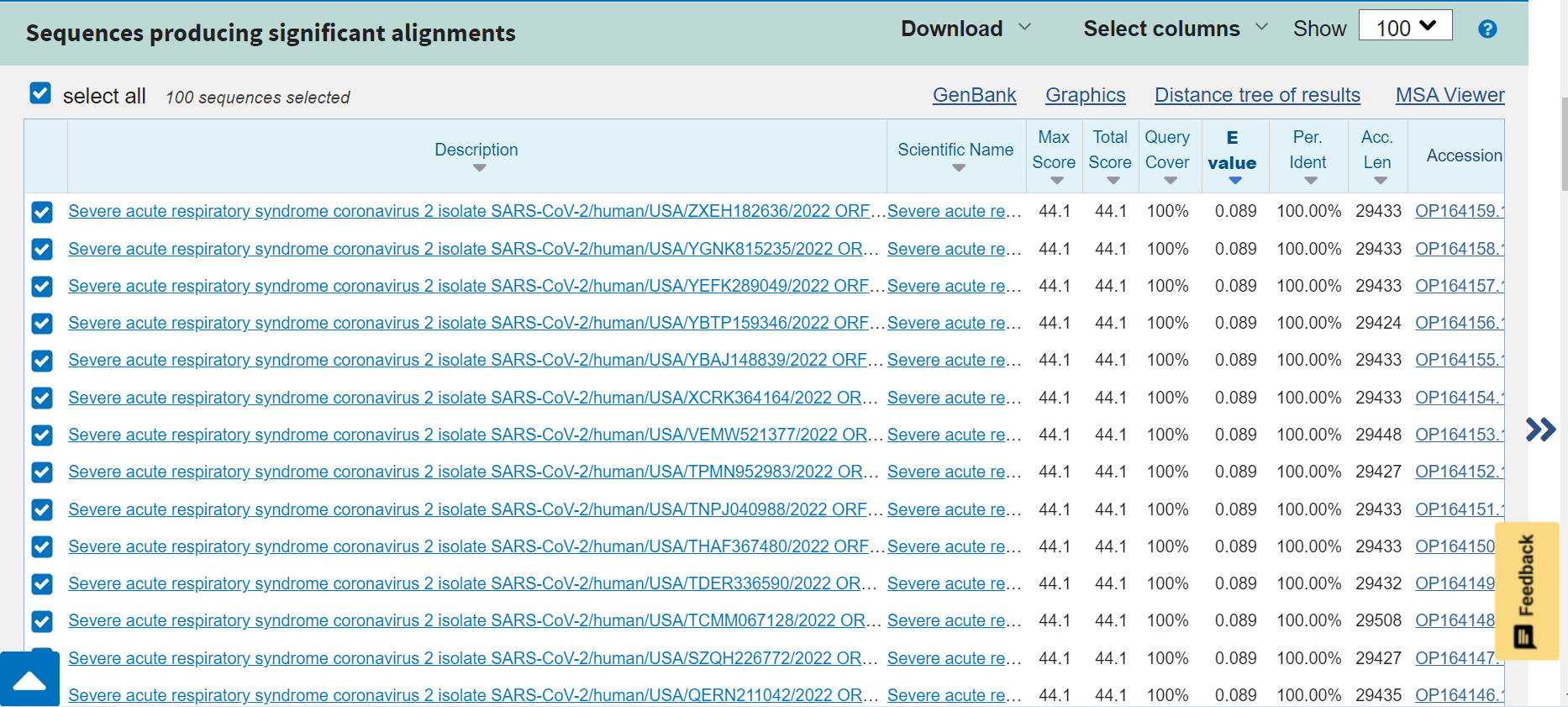

Supplement: Supplemental Information 2 [file peerj-10-14121-s002.zip › Supplemental 1-Blasting results of the LAMP primer sets/Set-2/LB primer.png]

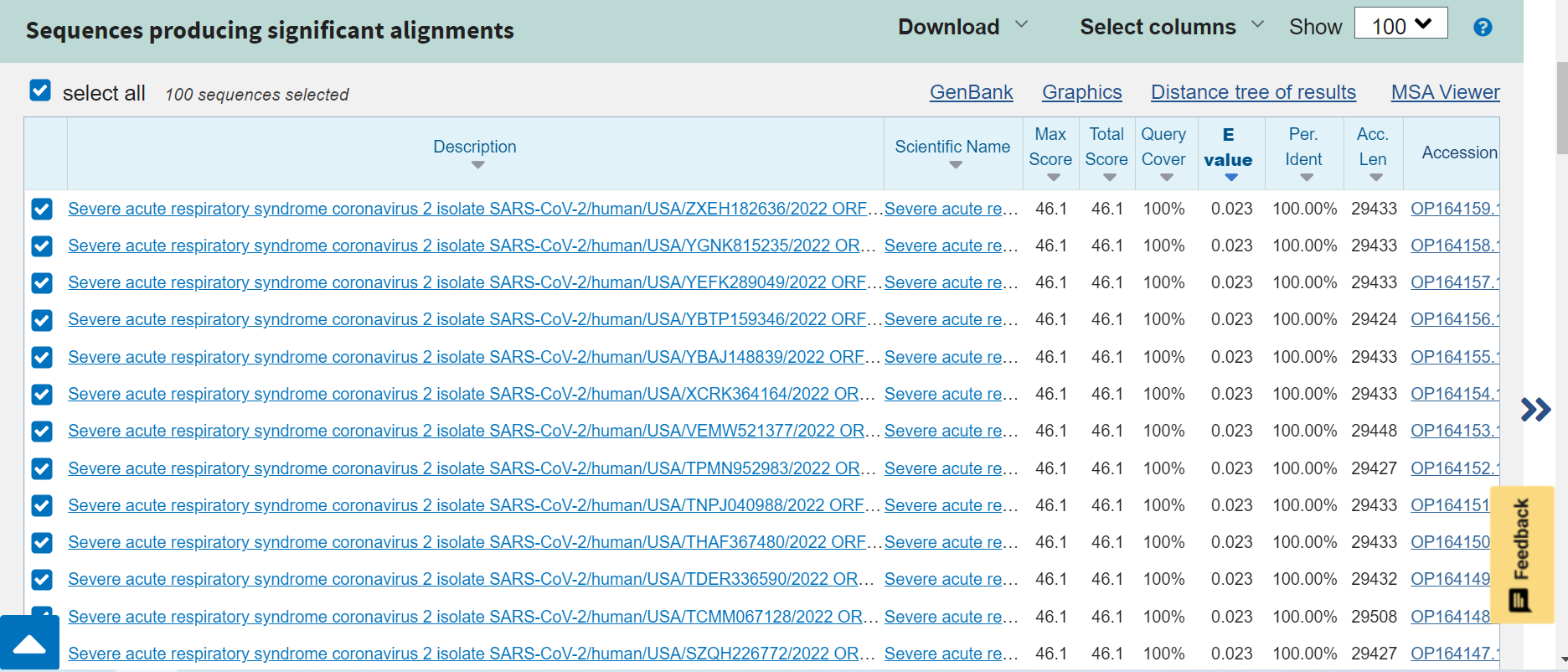

Supplement: Supplemental Information 2 [file peerj-10-14121-s002.zip › Supplemental 1-Blasting results of the LAMP primer sets/Set-2/LF primer.png]

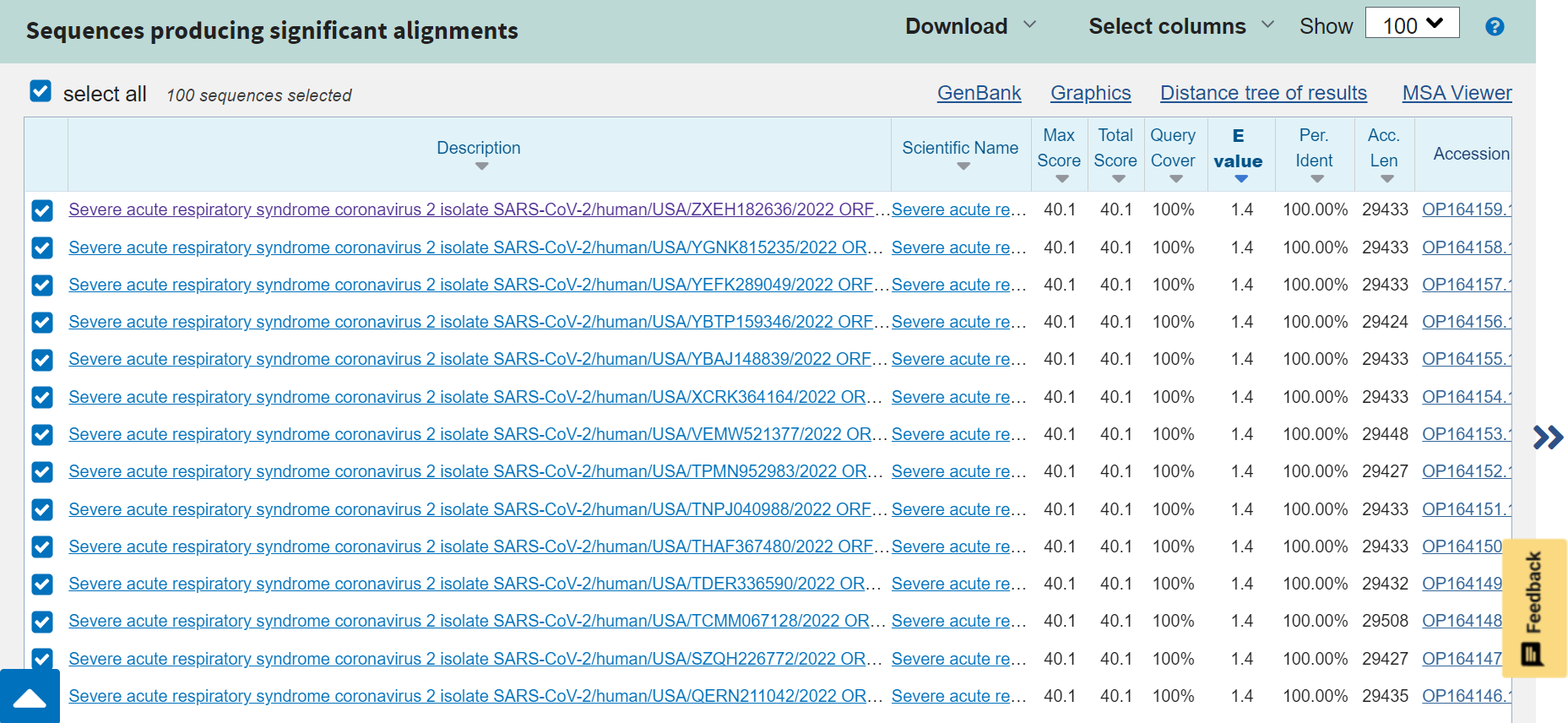

Supplement: Supplemental Information 2 [file peerj-10-14121-s002.zip › Supplemental 1-Blasting results of the LAMP primer sets/Set-3/B3 primer.png]

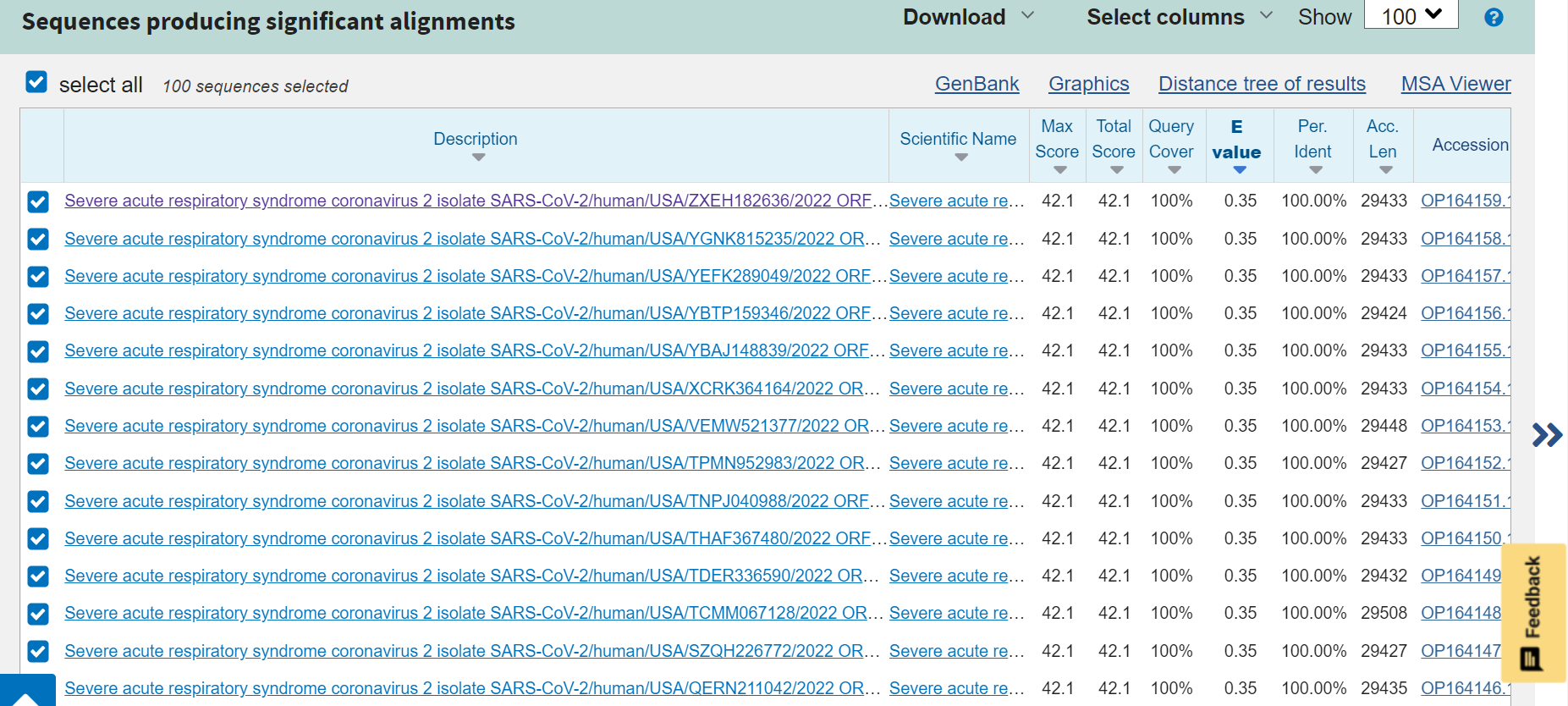

Supplement: Supplemental Information 2 [file peerj-10-14121-s002.zip › Supplemental 1-Blasting results of the LAMP primer sets/Set-3/BIP primer-B1c.png]

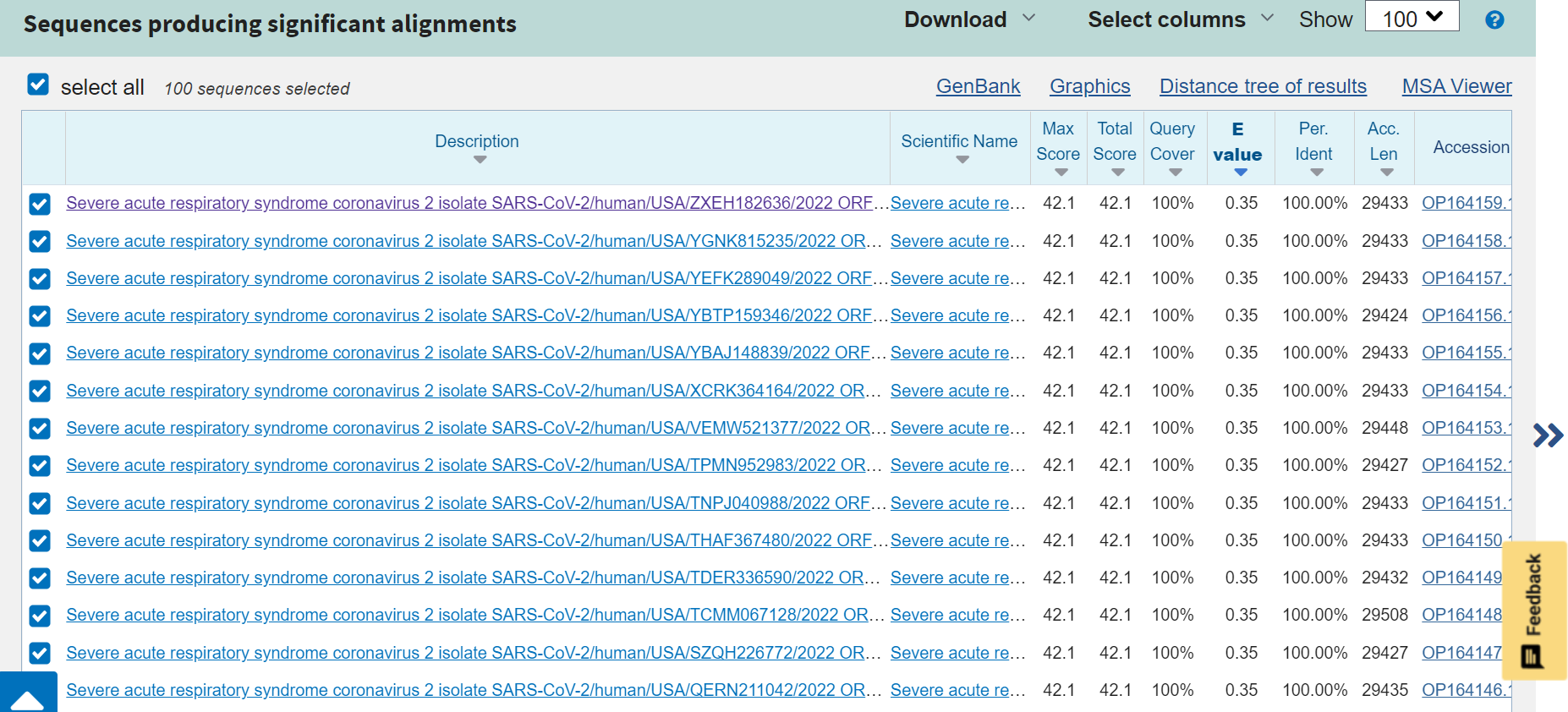

Supplement: Supplemental Information 2 [file peerj-10-14121-s002.zip › Supplemental 1-Blasting results of the LAMP primer sets/Set-3/BIP primer-B2.png]

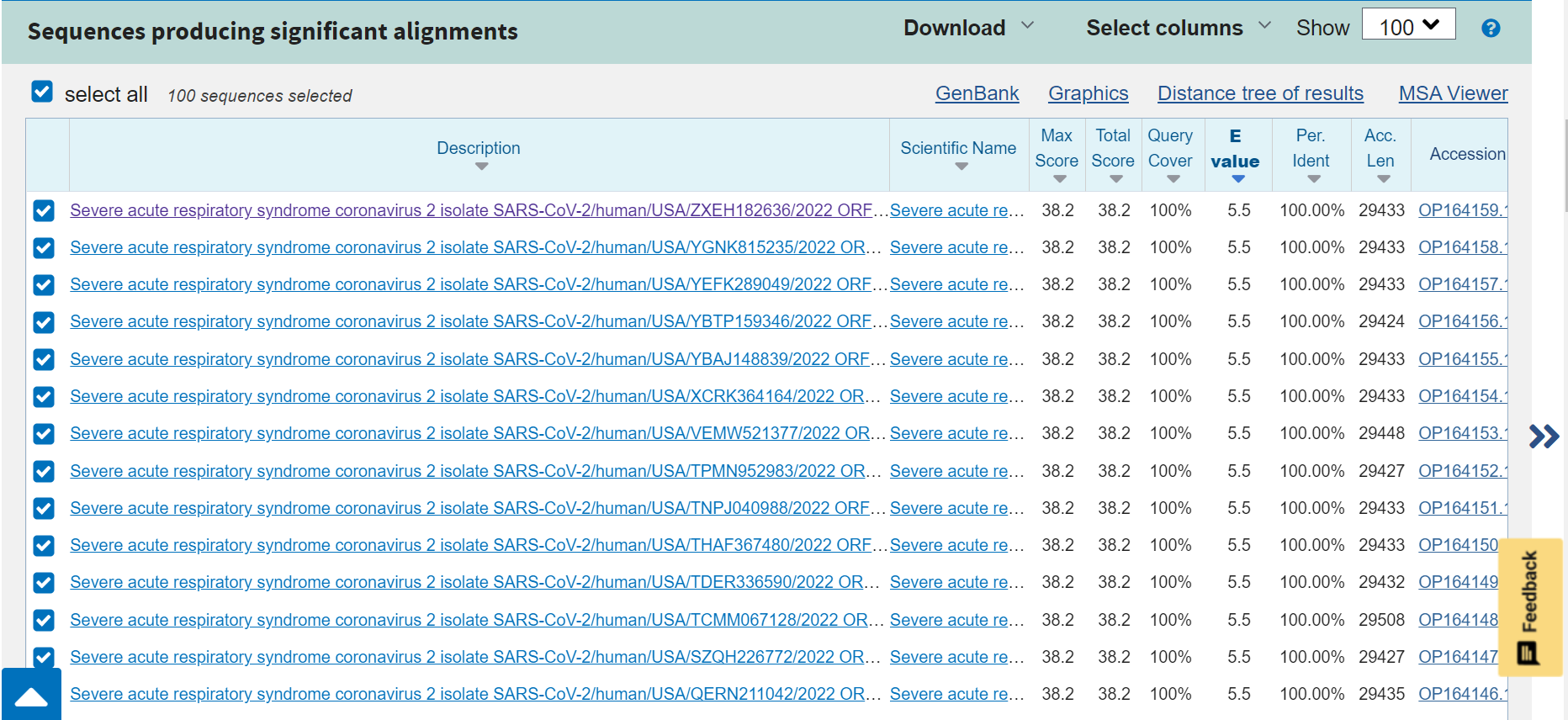

Supplement: Supplemental Information 2 [file peerj-10-14121-s002.zip › Supplemental 1-Blasting results of the LAMP primer sets/Set-3/F3 primer.png]

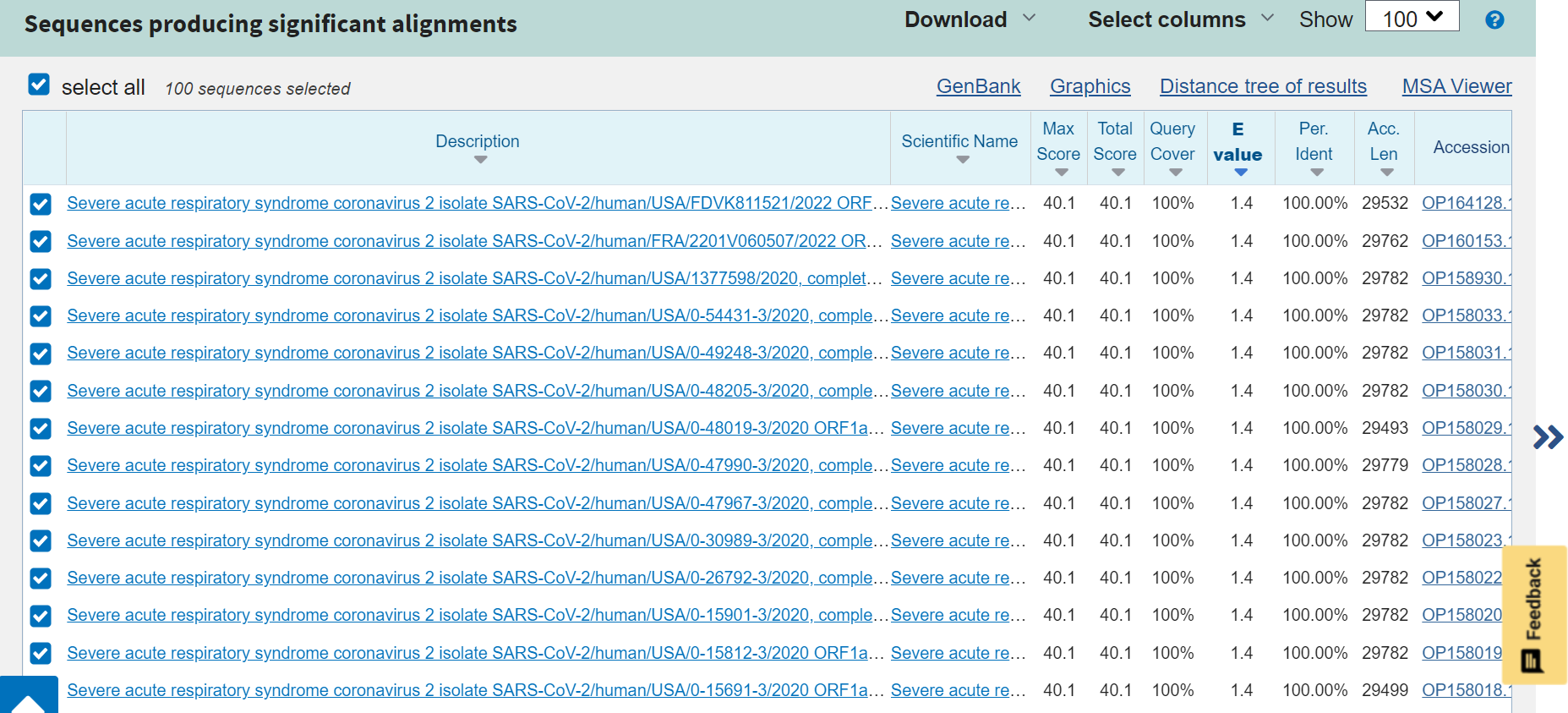

Supplement: Supplemental Information 2 [file peerj-10-14121-s002.zip › Supplemental 1-Blasting results of the LAMP primer sets/Set-3/FIP primer-F1c.png]

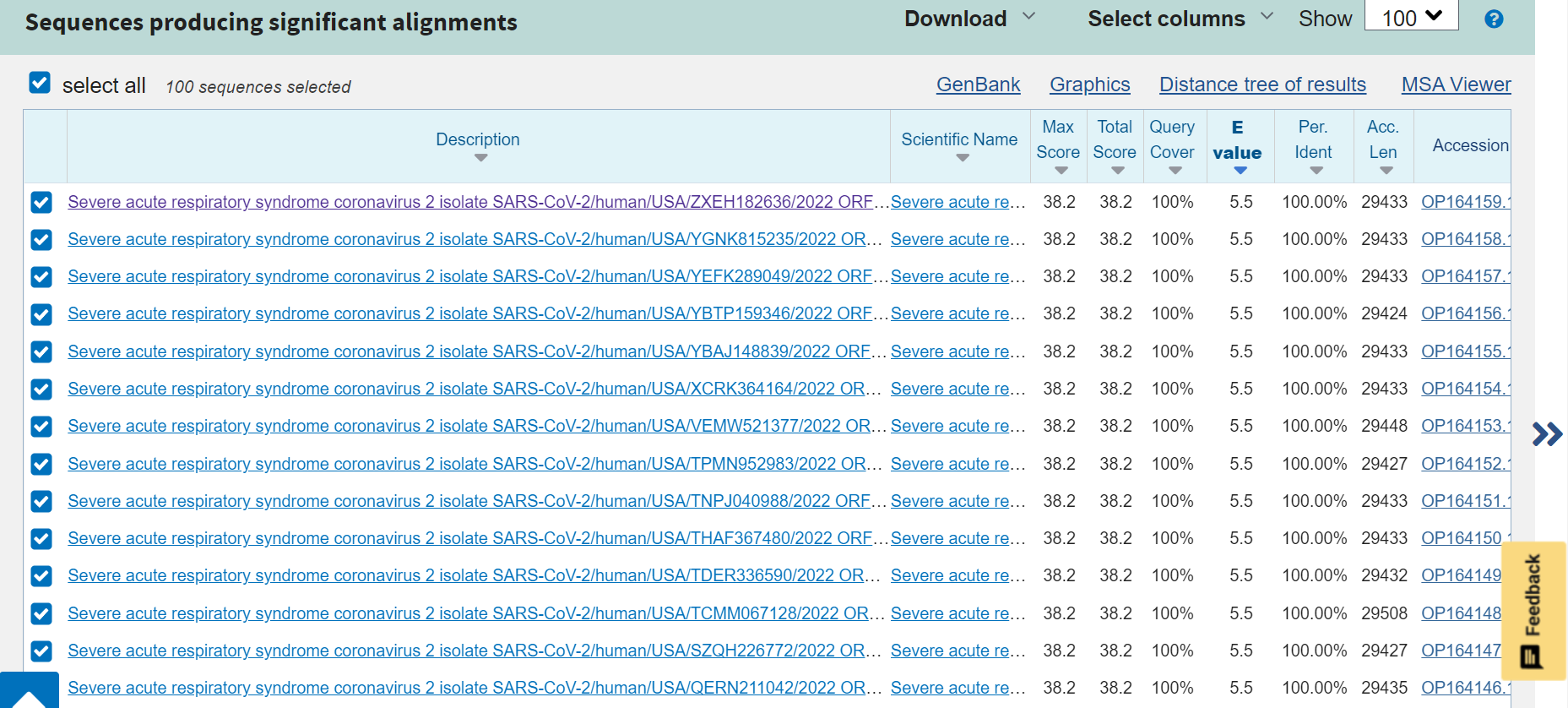

Supplement: Supplemental Information 2 [file peerj-10-14121-s002.zip › Supplemental 1-Blasting results of the LAMP primer sets/Set-3/FIP primer-F2.png]

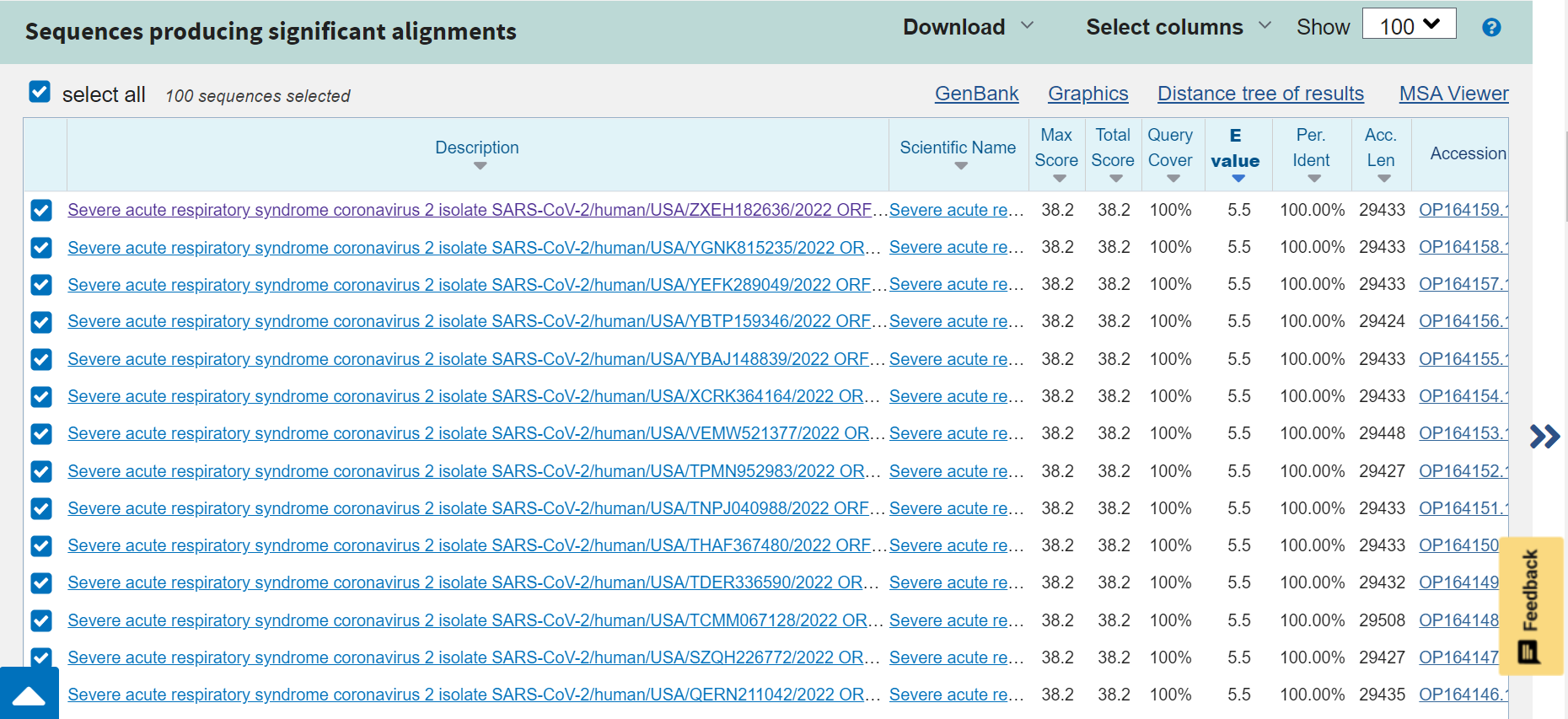

Supplement: Supplemental Information 2 [file peerj-10-14121-s002.zip › Supplemental 1-Blasting results of the LAMP primer sets/Set-3/LB primer.png]

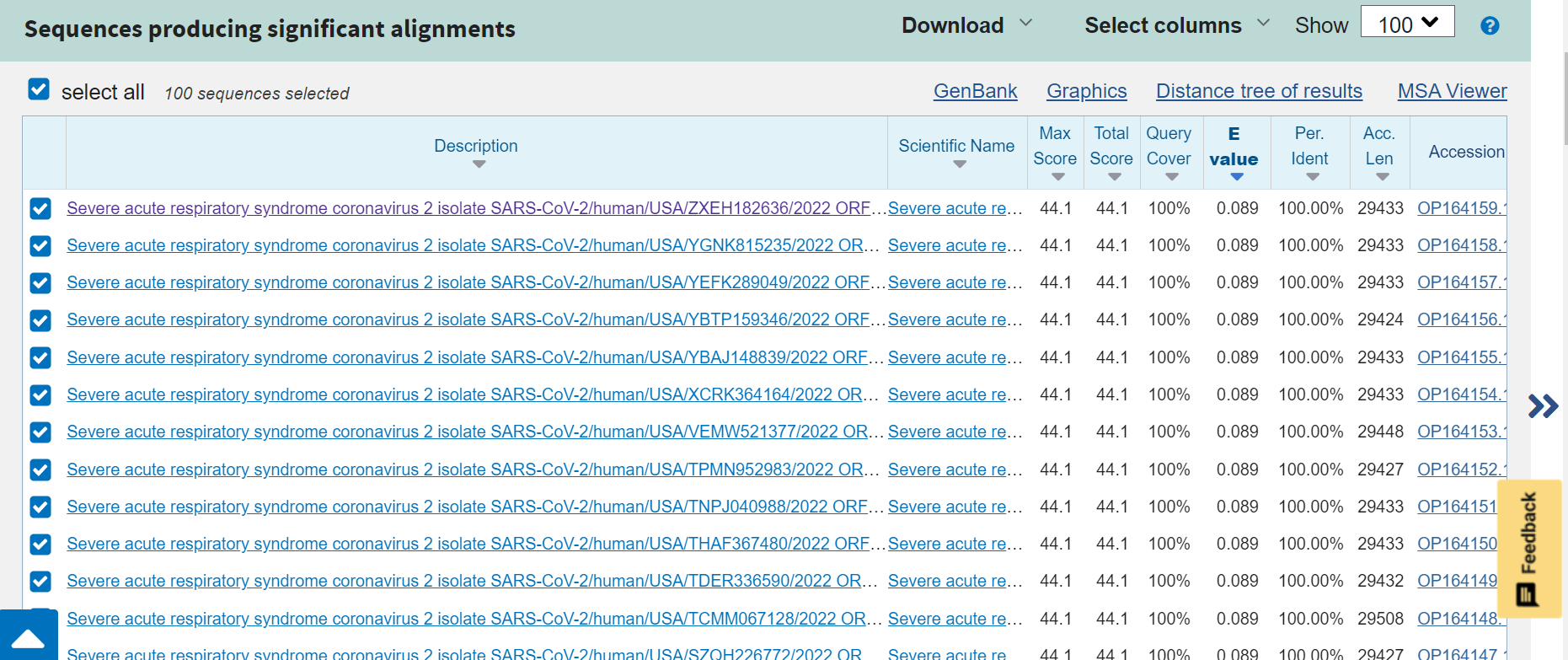

Supplement: Supplemental Information 2 [file peerj-10-14121-s002.zip › Supplemental 1-Blasting results of the LAMP primer sets/Set-3/LF primer.png]

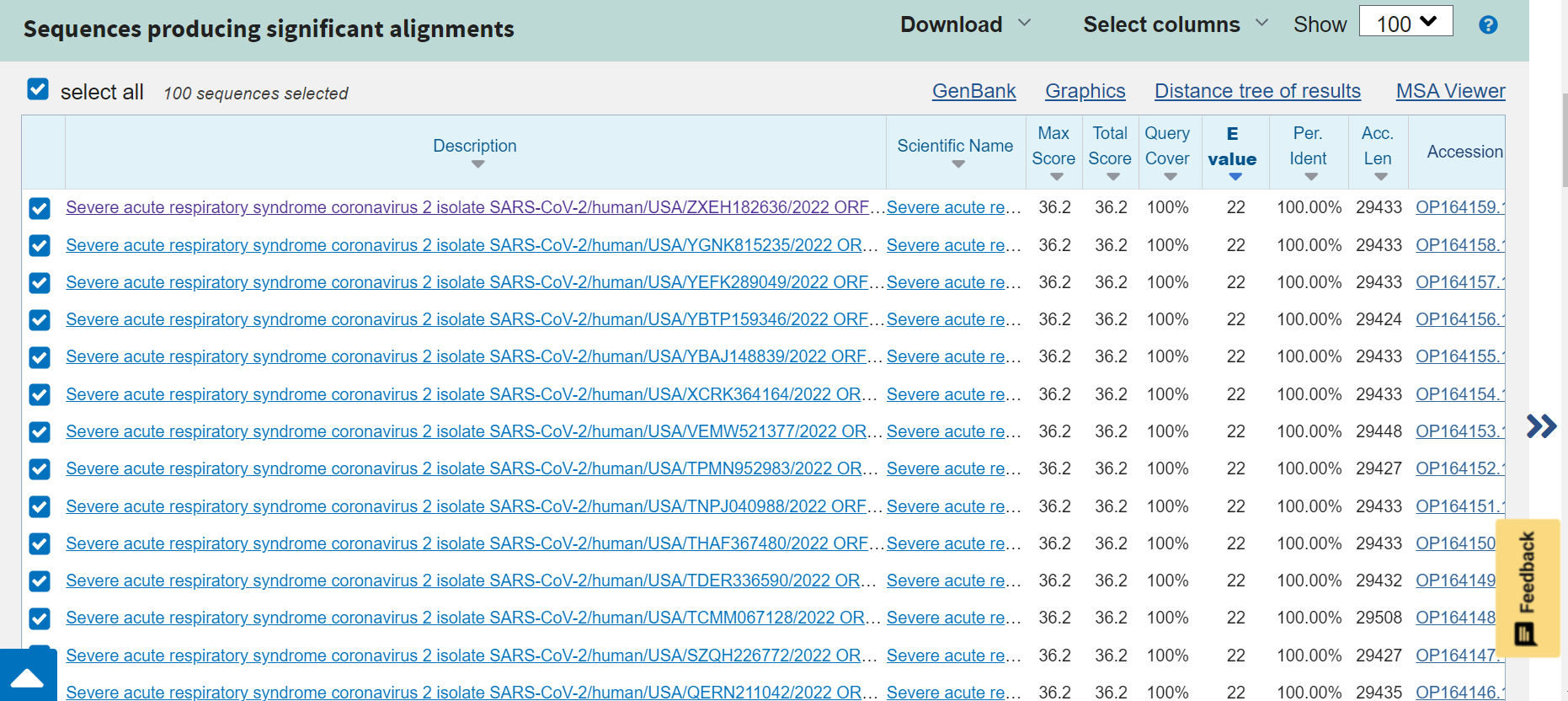

Supplement: Supplemental Information 2 [file peerj-10-14121-s002.zip › Supplemental 1-Blasting results of the LAMP primer sets/Set-4/B3 primer.png]

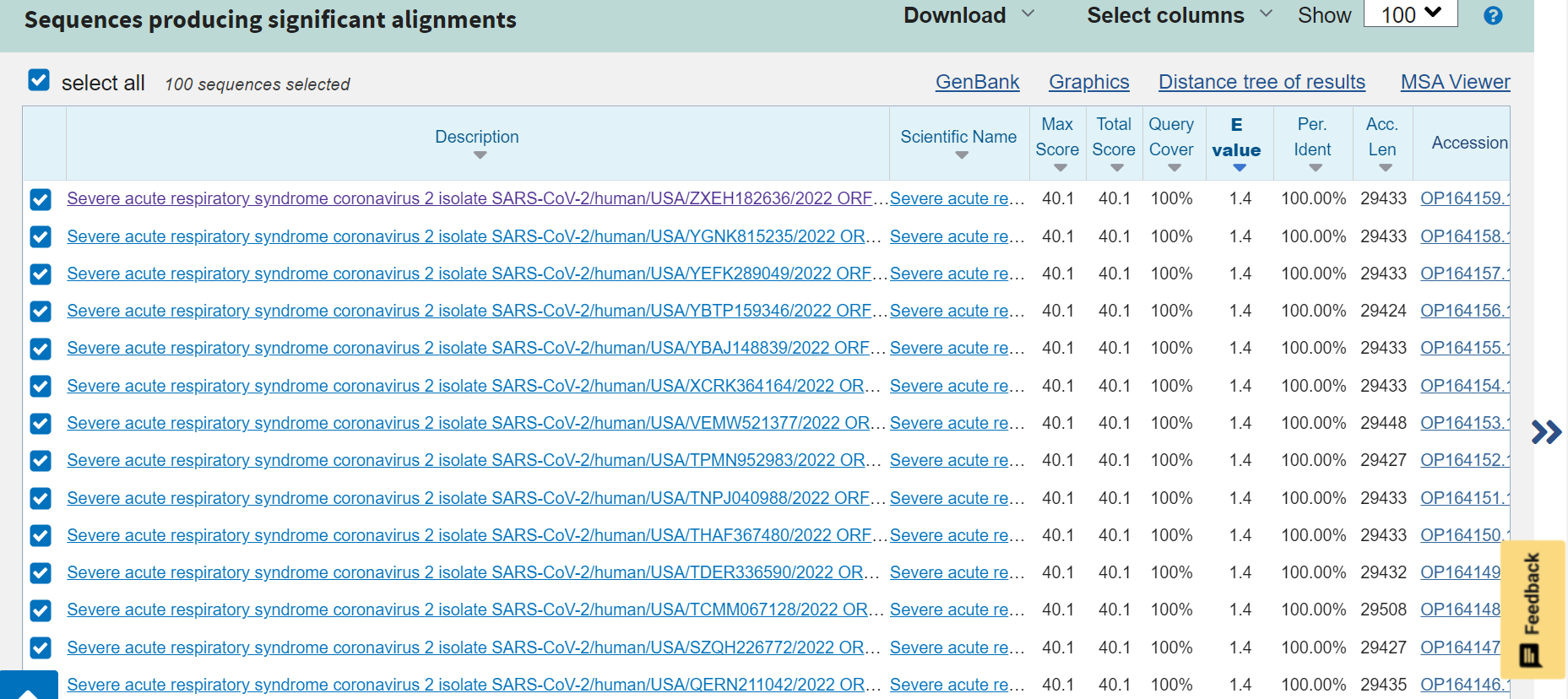

Supplement: Supplemental Information 2 [file peerj-10-14121-s002.zip › Supplemental 1-Blasting results of the LAMP primer sets/Set-4/BIP primer-B1c.png]

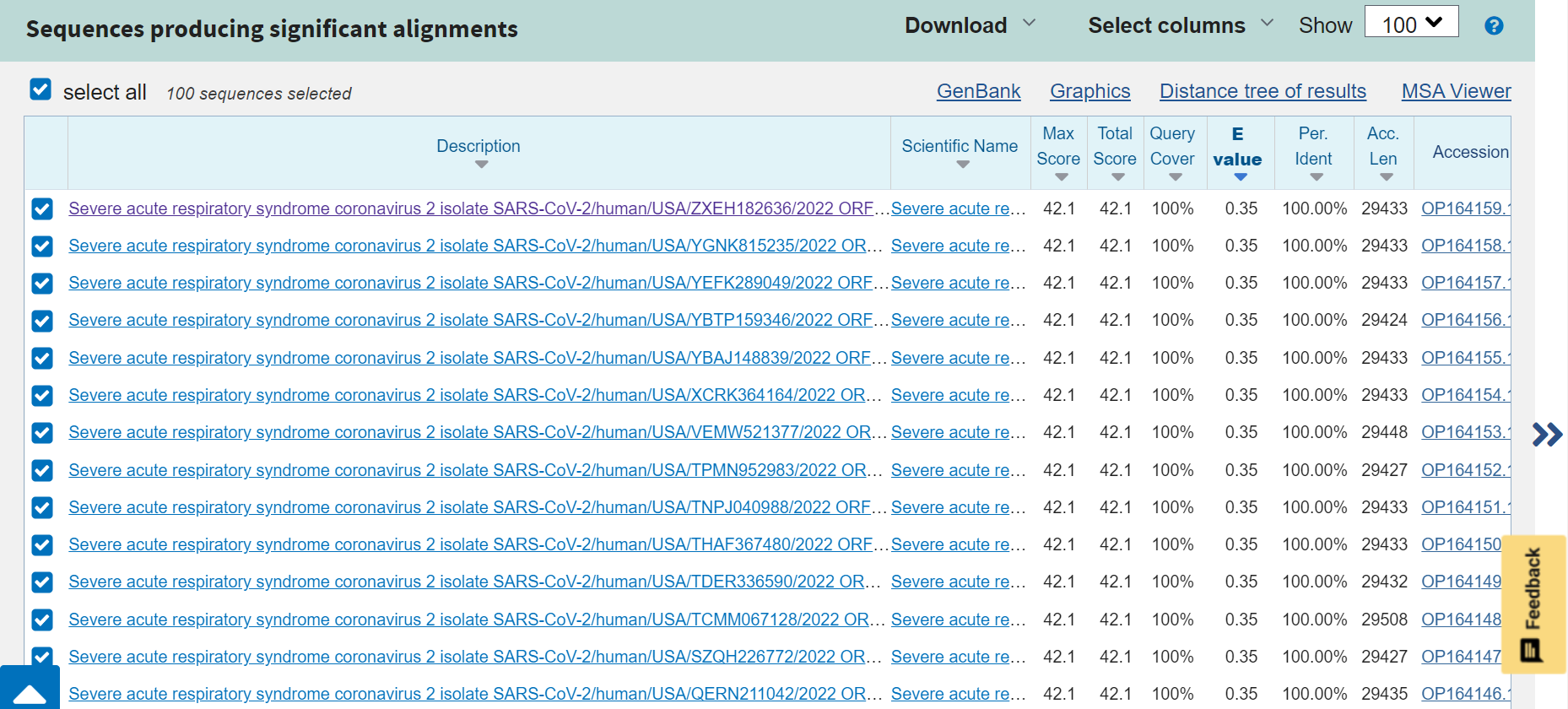

Supplement: Supplemental Information 2 [file peerj-10-14121-s002.zip › Supplemental 1-Blasting results of the LAMP primer sets/Set-4/BIP primer-B2.png]

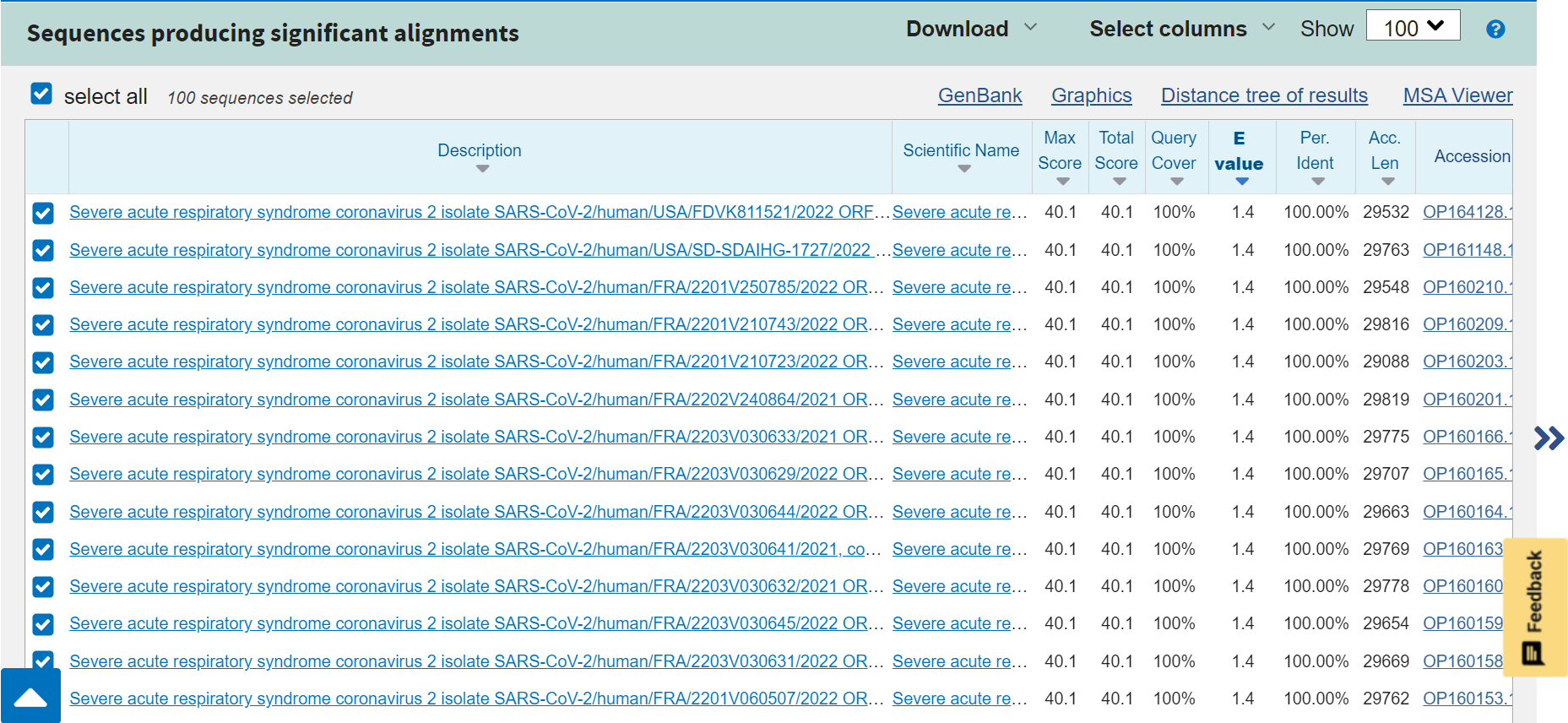

Supplement: Supplemental Information 2 [file peerj-10-14121-s002.zip › Supplemental 1-Blasting results of the LAMP primer sets/Set-4/F3 primer.png]

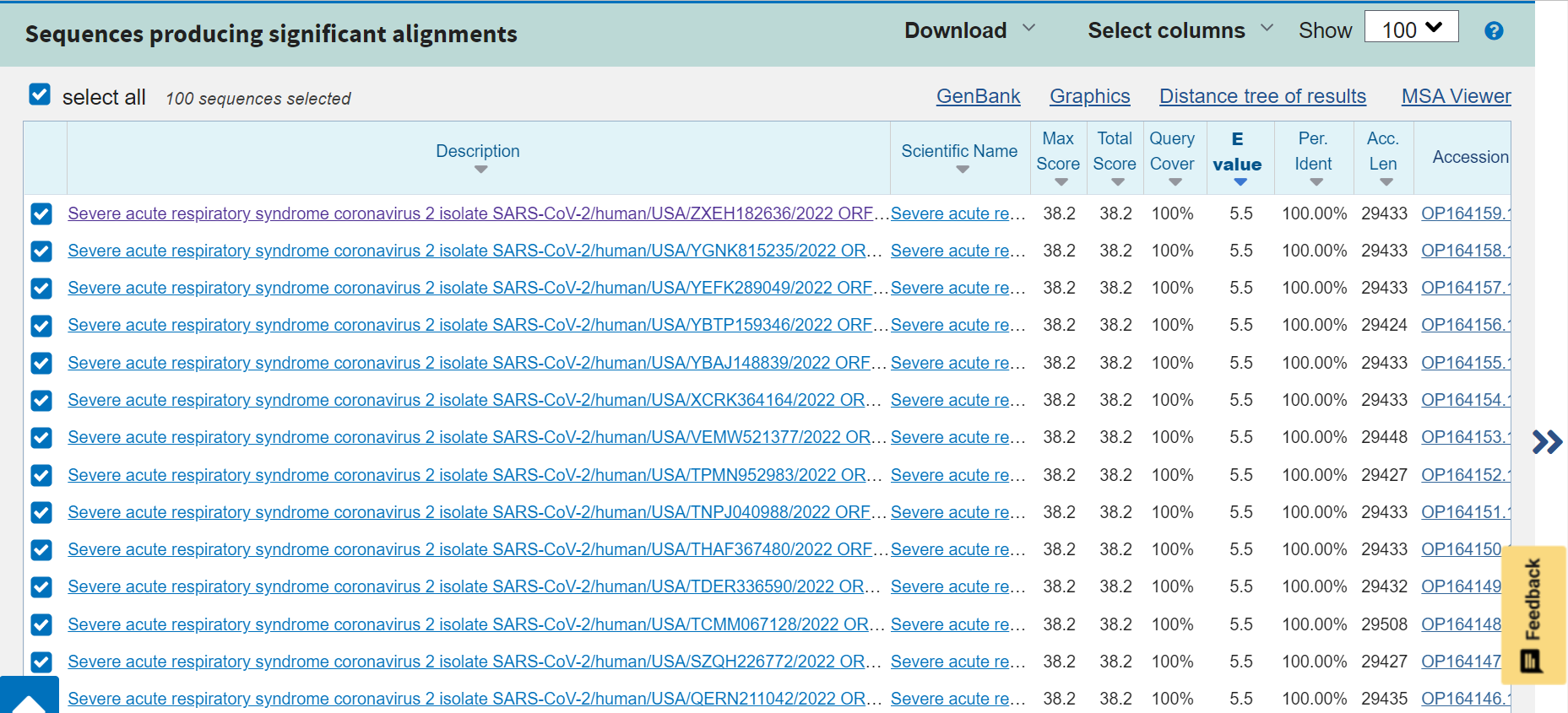

Supplement: Supplemental Information 2 [file peerj-10-14121-s002.zip › Supplemental 1-Blasting results of the LAMP primer sets/Set-4/FIP primer-F1c.png]

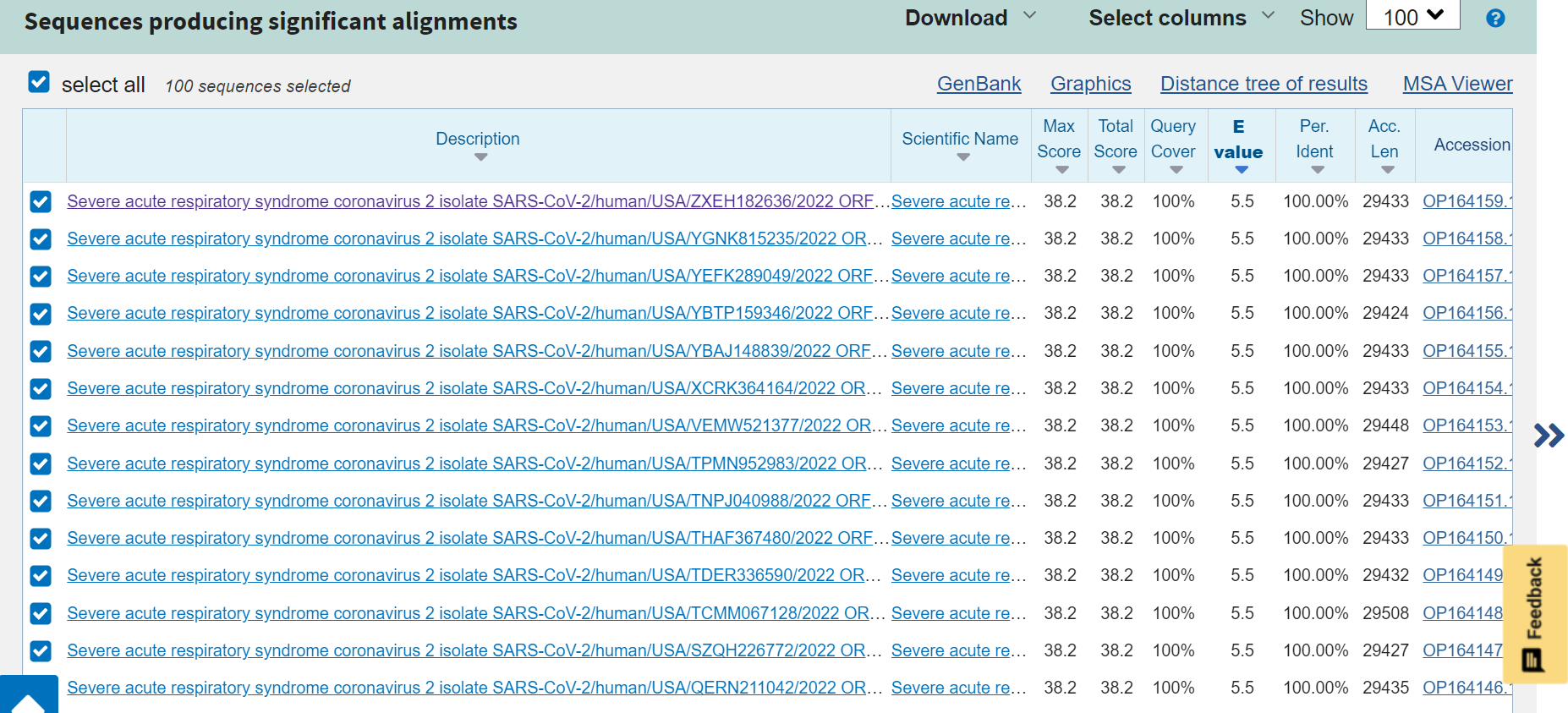

Supplement: Supplemental Information 2 [file peerj-10-14121-s002.zip › Supplemental 1-Blasting results of the LAMP primer sets/Set-4/FIP primer-F2.png]

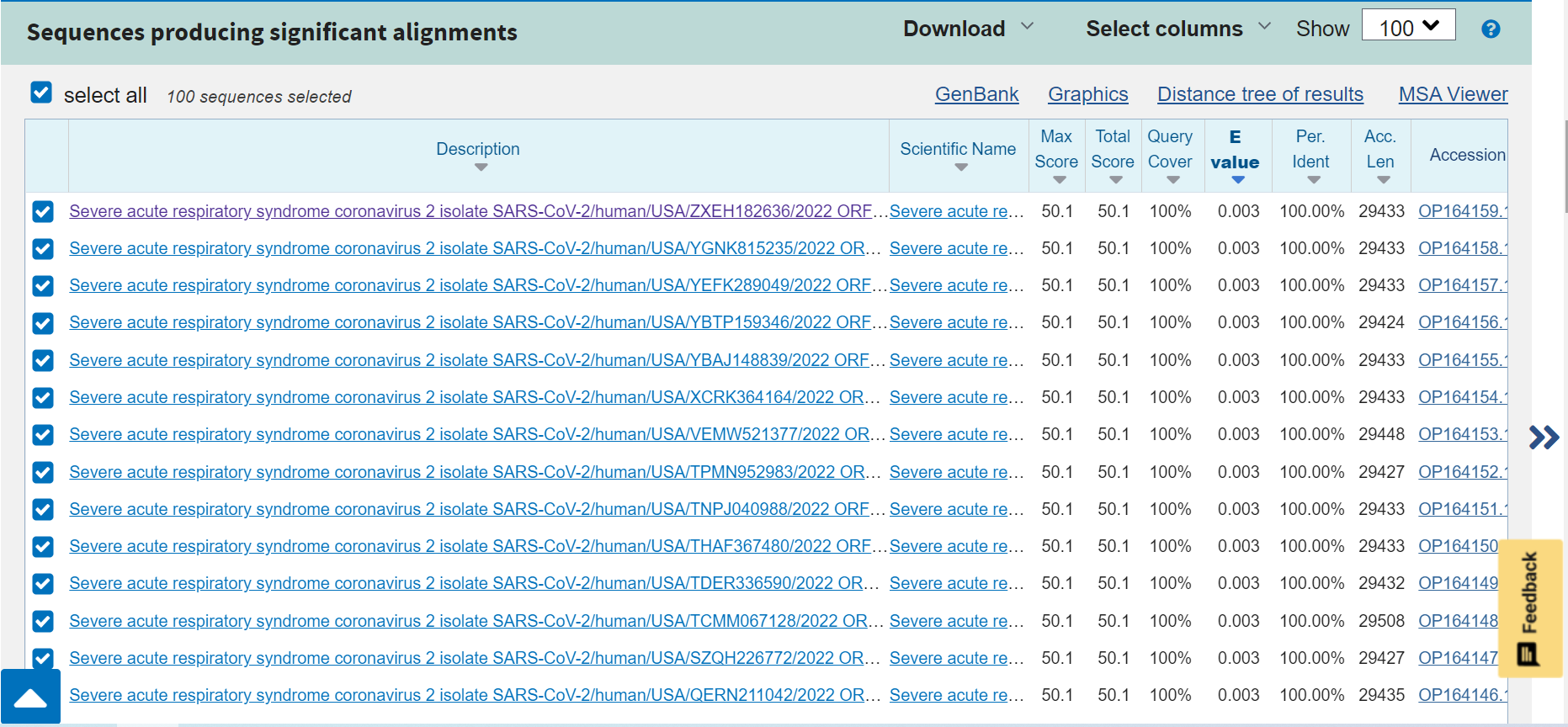

Supplement: Supplemental Information 2 [file peerj-10-14121-s002.zip › Supplemental 1-Blasting results of the LAMP primer sets/Set-4/LB primer.png]

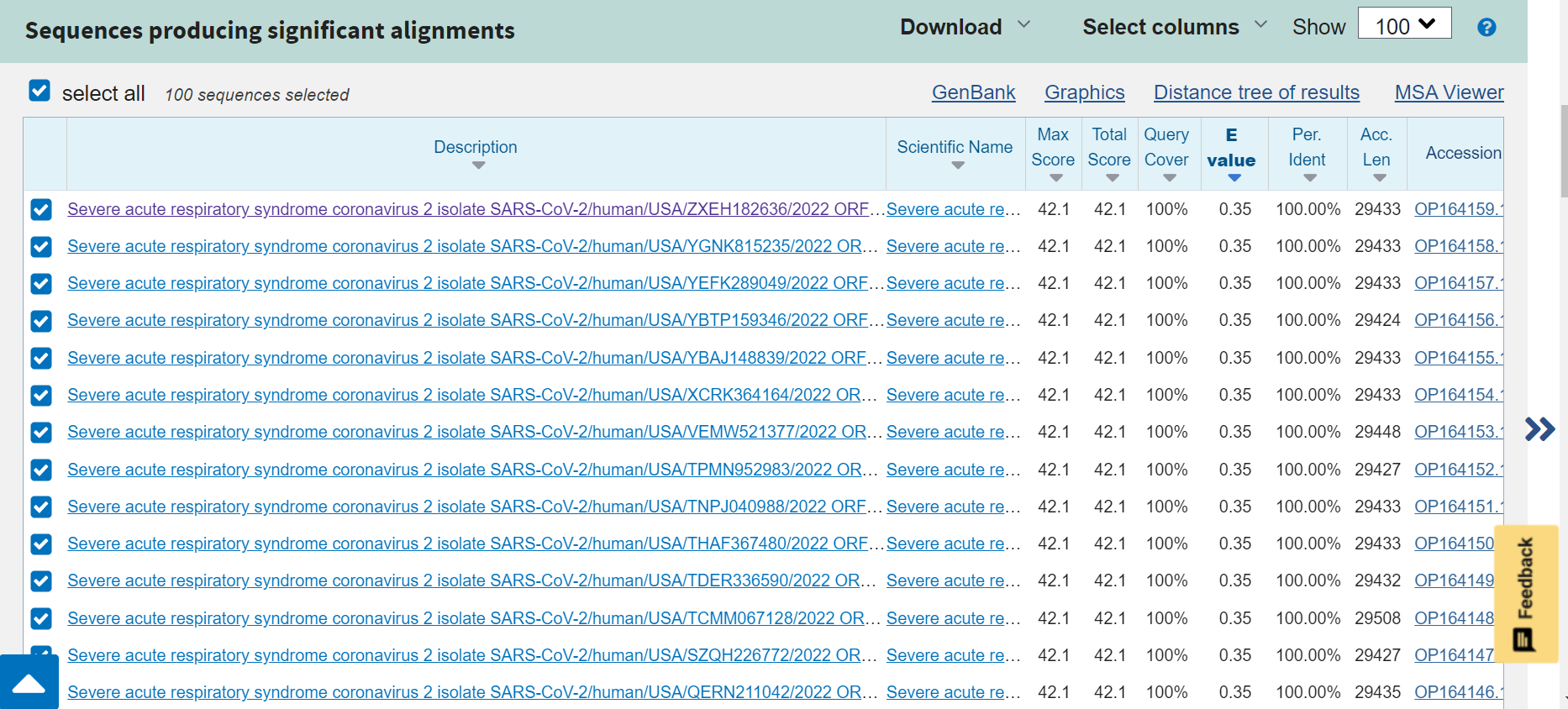

Supplement: Supplemental Information 2 [file peerj-10-14121-s002.zip › Supplemental 1-Blasting results of the LAMP primer sets/Set-4/LF primer.png]

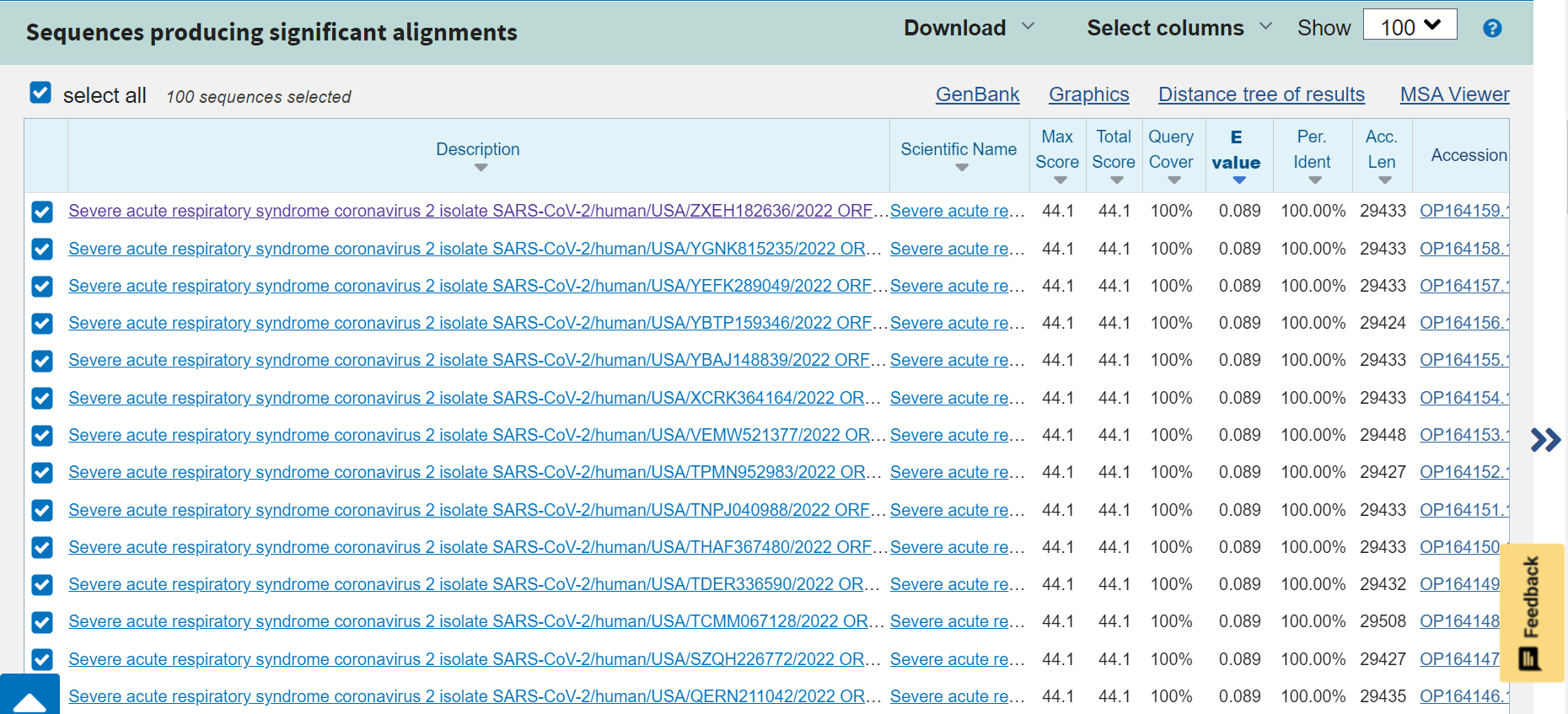

Supplement: Supplemental Information 2 [file peerj-10-14121-s002.zip › Supplemental 1-Blasting results of the LAMP primer sets/Set-5/B3 primer.png]

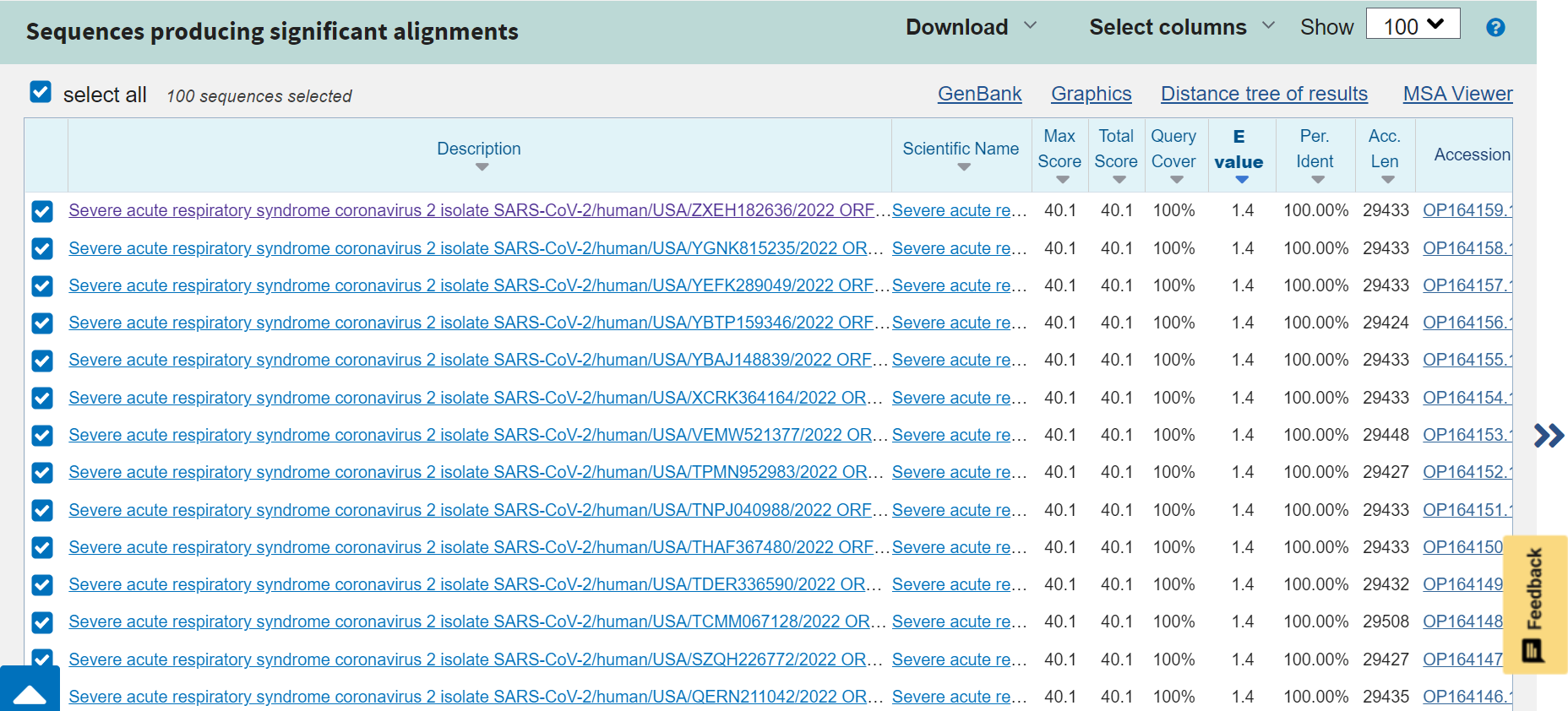

Supplement: Supplemental Information 2 [file peerj-10-14121-s002.zip › Supplemental 1-Blasting results of the LAMP primer sets/Set-5/BIP primer-B1c.png]

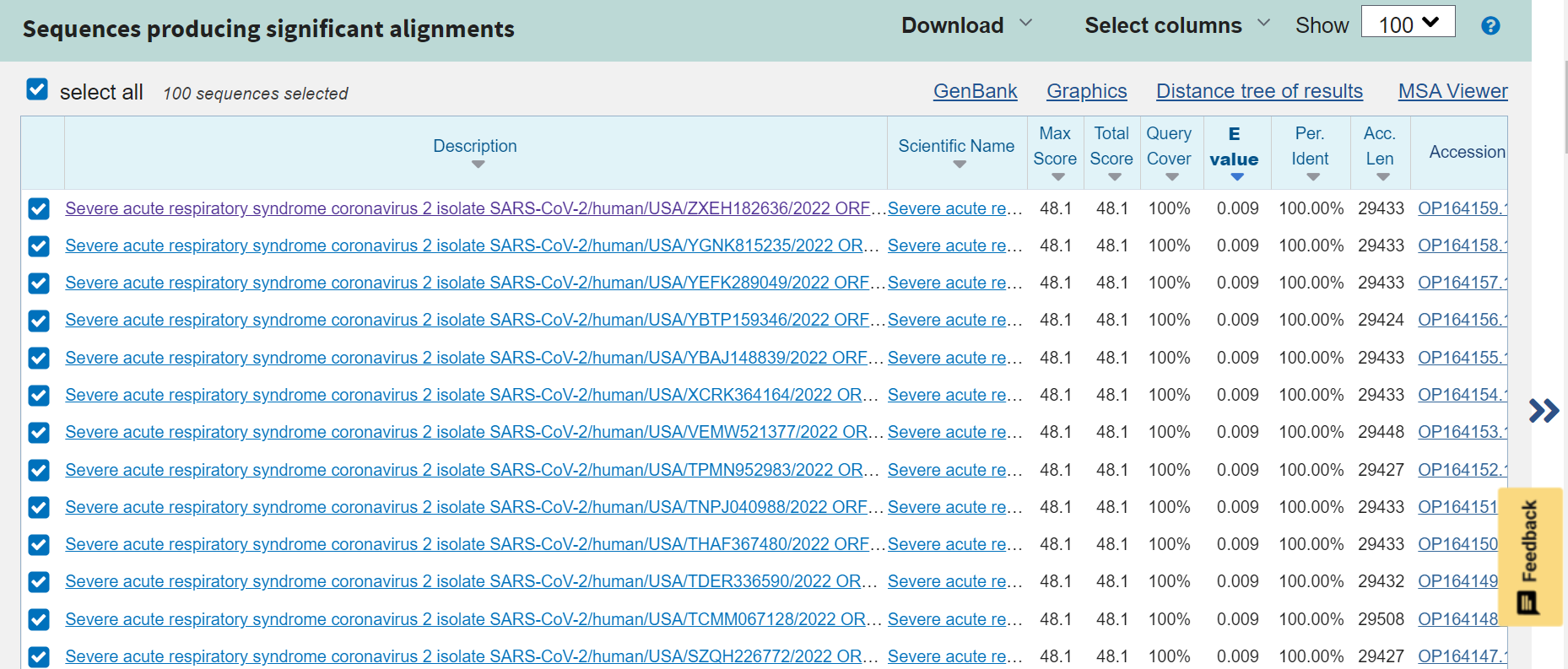

Supplement: Supplemental Information 2 [file peerj-10-14121-s002.zip › Supplemental 1-Blasting results of the LAMP primer sets/Set-5/BIP primer-B2.png]

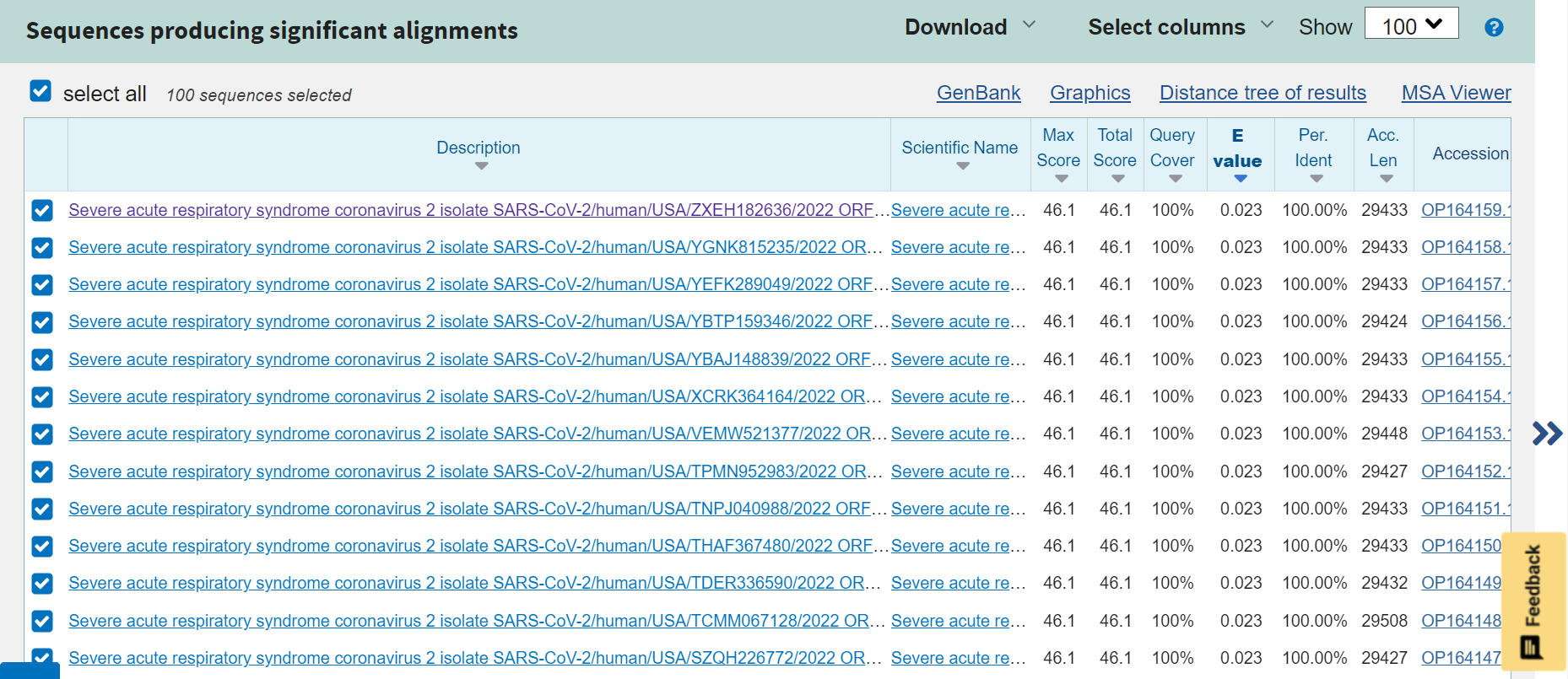

Supplement: Supplemental Information 2 [file peerj-10-14121-s002.zip › Supplemental 1-Blasting results of the LAMP primer sets/Set-5/F3 primer.png]

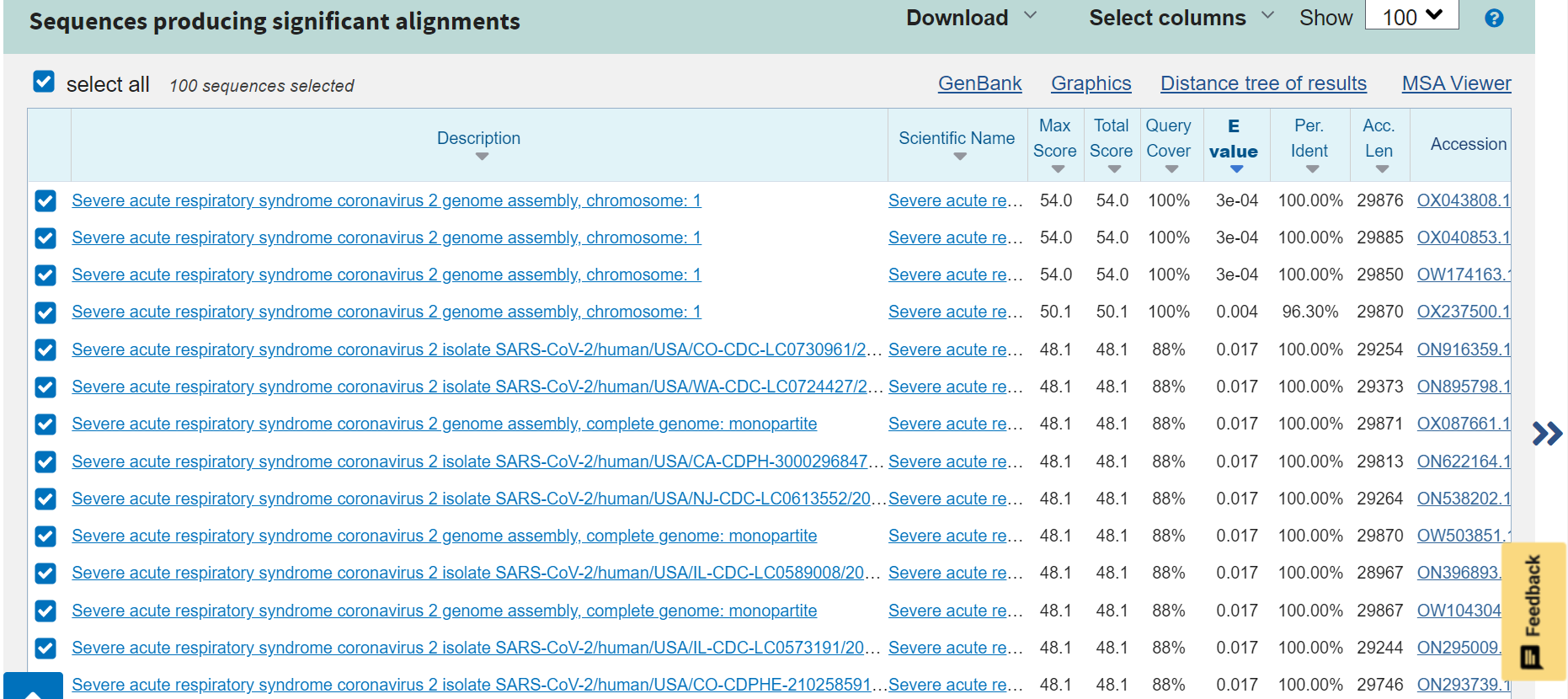

Supplement: Supplemental Information 2 [file peerj-10-14121-s002.zip › Supplemental 1-Blasting results of the LAMP primer sets/Set-5/FIP primer-F1c.png]

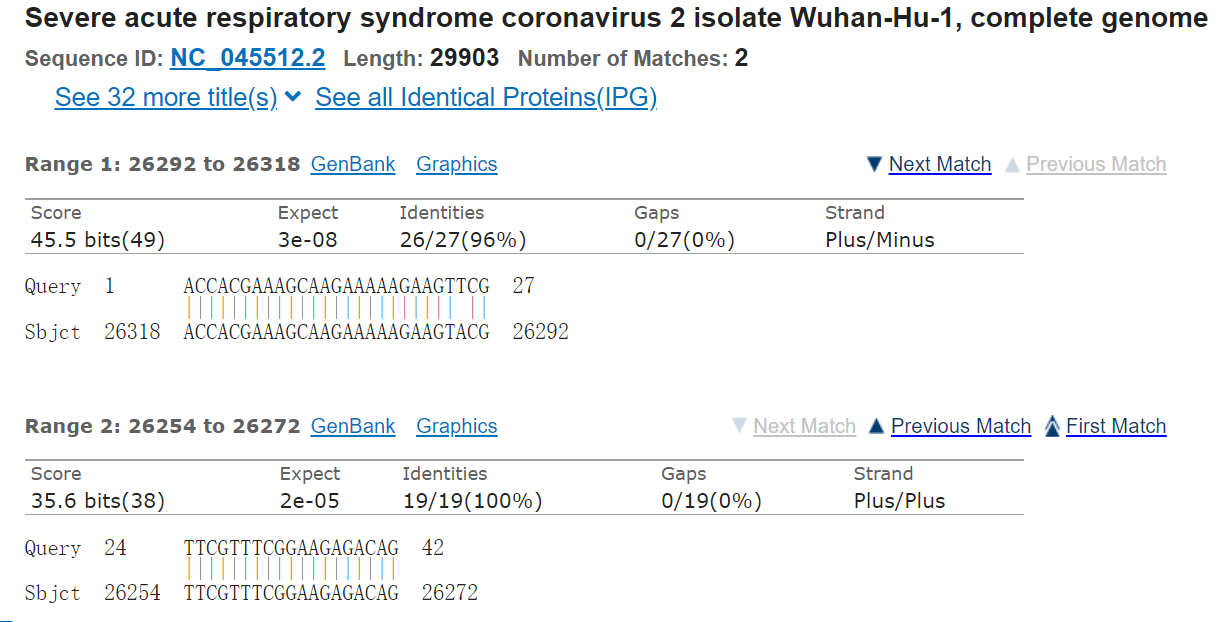

Supplement: Supplemental Information 2 [file peerj-10-14121-s002.zip › Supplemental 1-Blasting results of the LAMP primer sets/Set-5/FIP primer-F2.png]

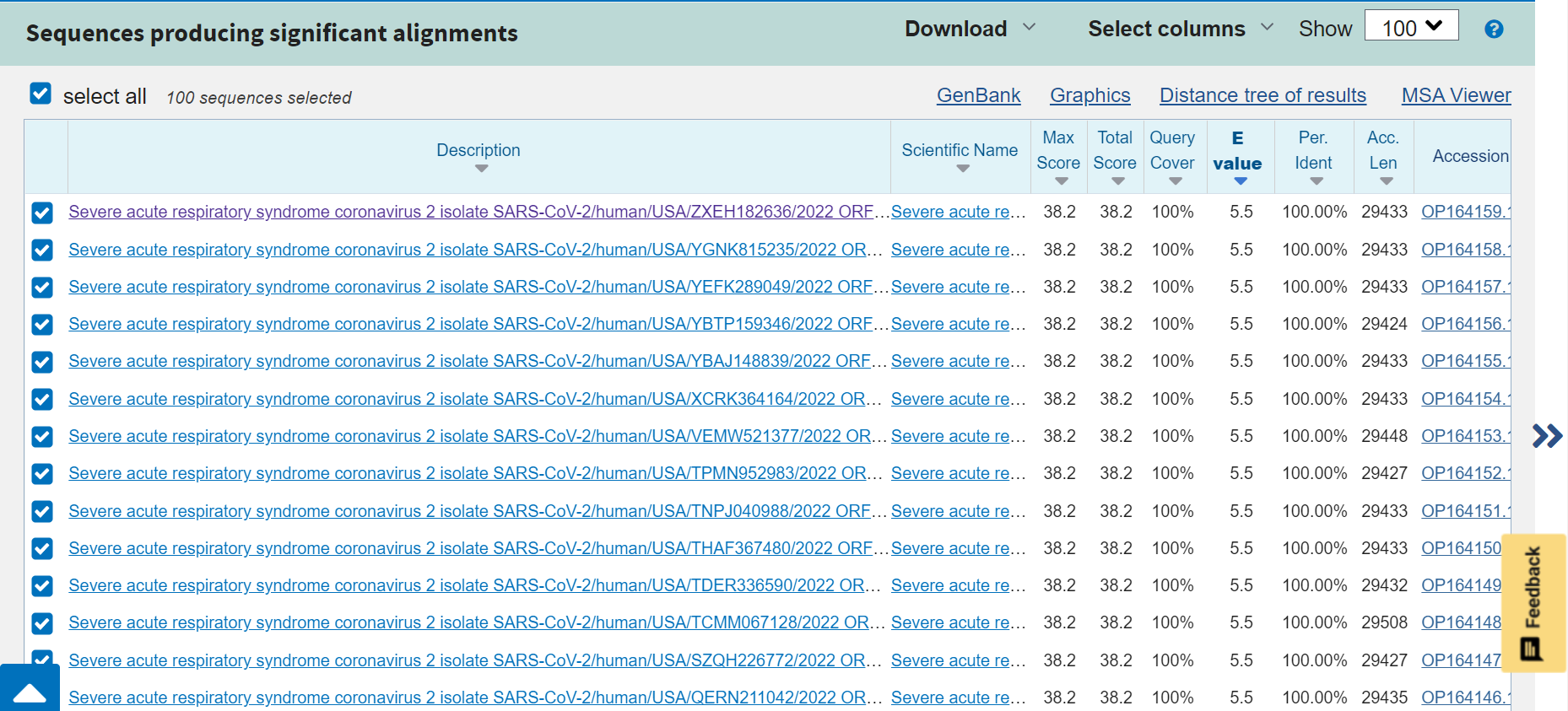

Supplement: Supplemental Information 2 [file peerj-10-14121-s002.zip › Supplemental 1-Blasting results of the LAMP primer sets/Set-5/LB primer.png]

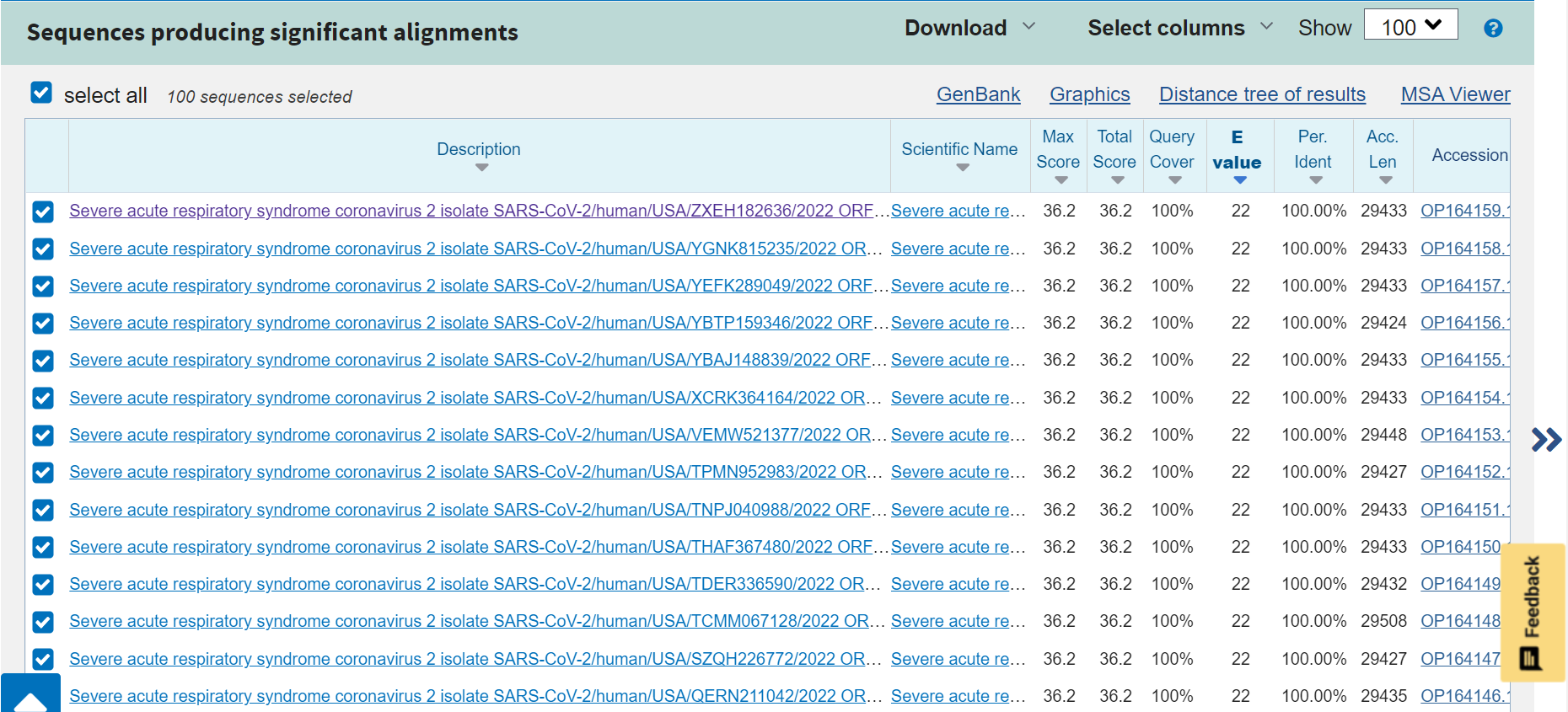

Supplement: Supplemental Information 2 [file peerj-10-14121-s002.zip › Supplemental 1-Blasting results of the LAMP primer sets/Set-5/LF primer.png]

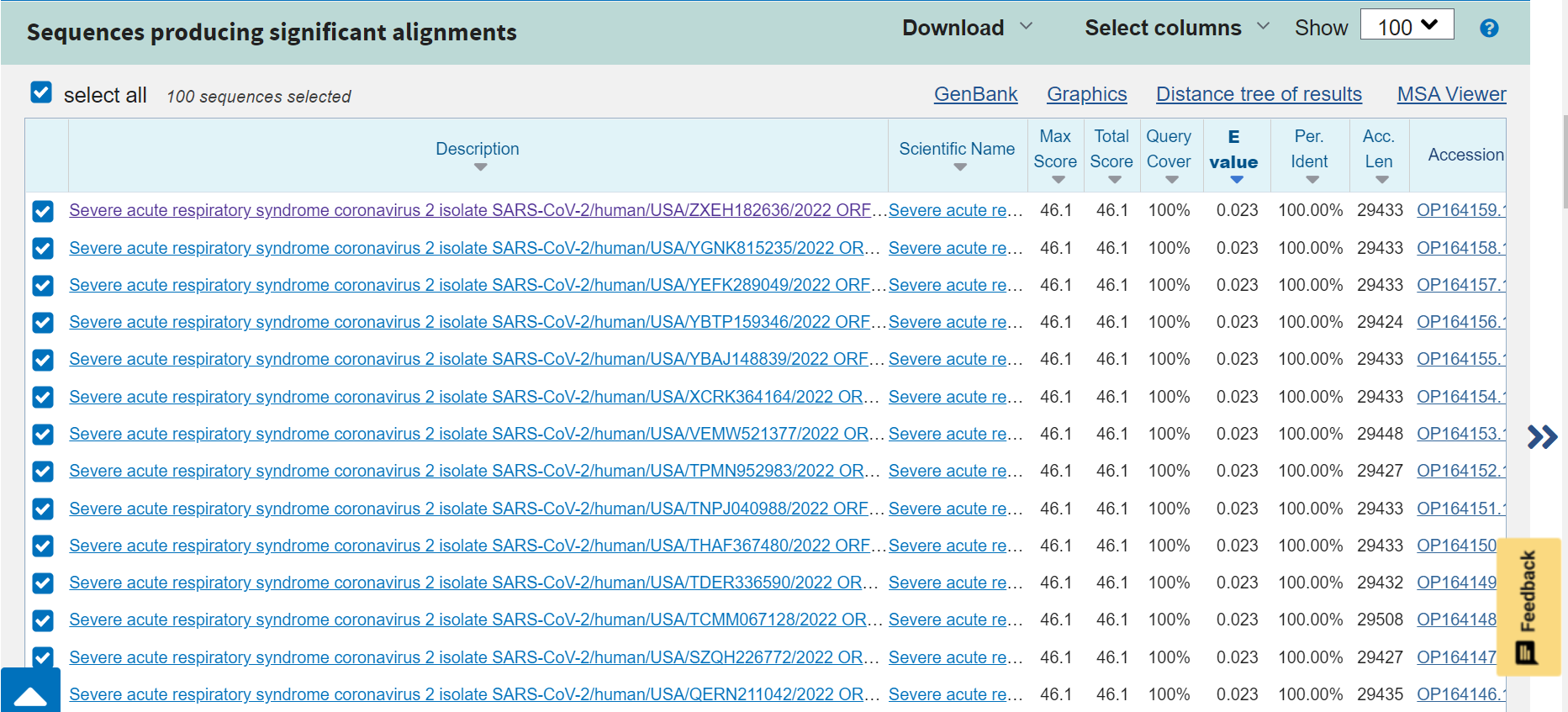

Supplement: Supplemental Information 2 [file peerj-10-14121-s002.zip › Supplemental 1-Blasting results of the LAMP primer sets/Set-6/B3 primer.png]

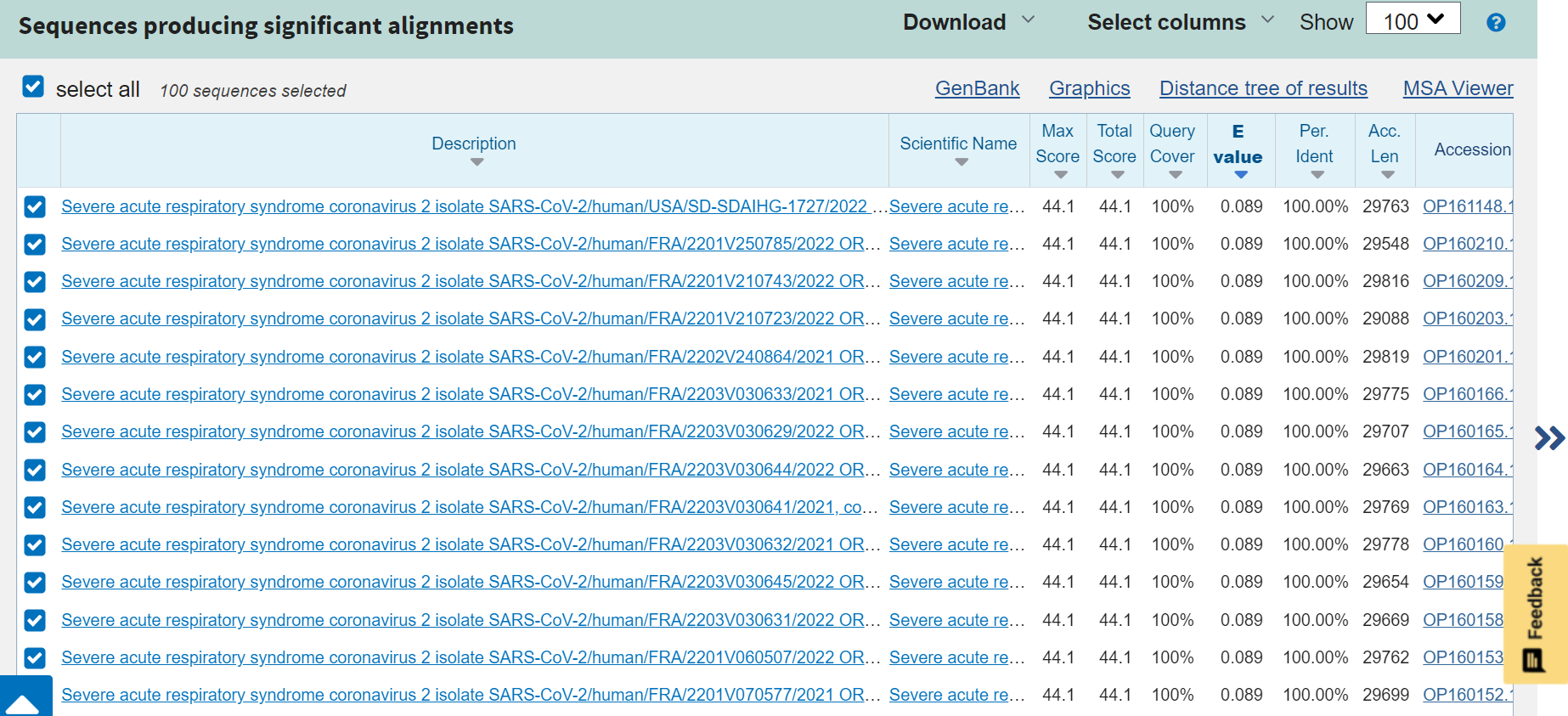

Supplement: Supplemental Information 2 [file peerj-10-14121-s002.zip › Supplemental 1-Blasting results of the LAMP primer sets/Set-6/BIP primer-B1c.png]

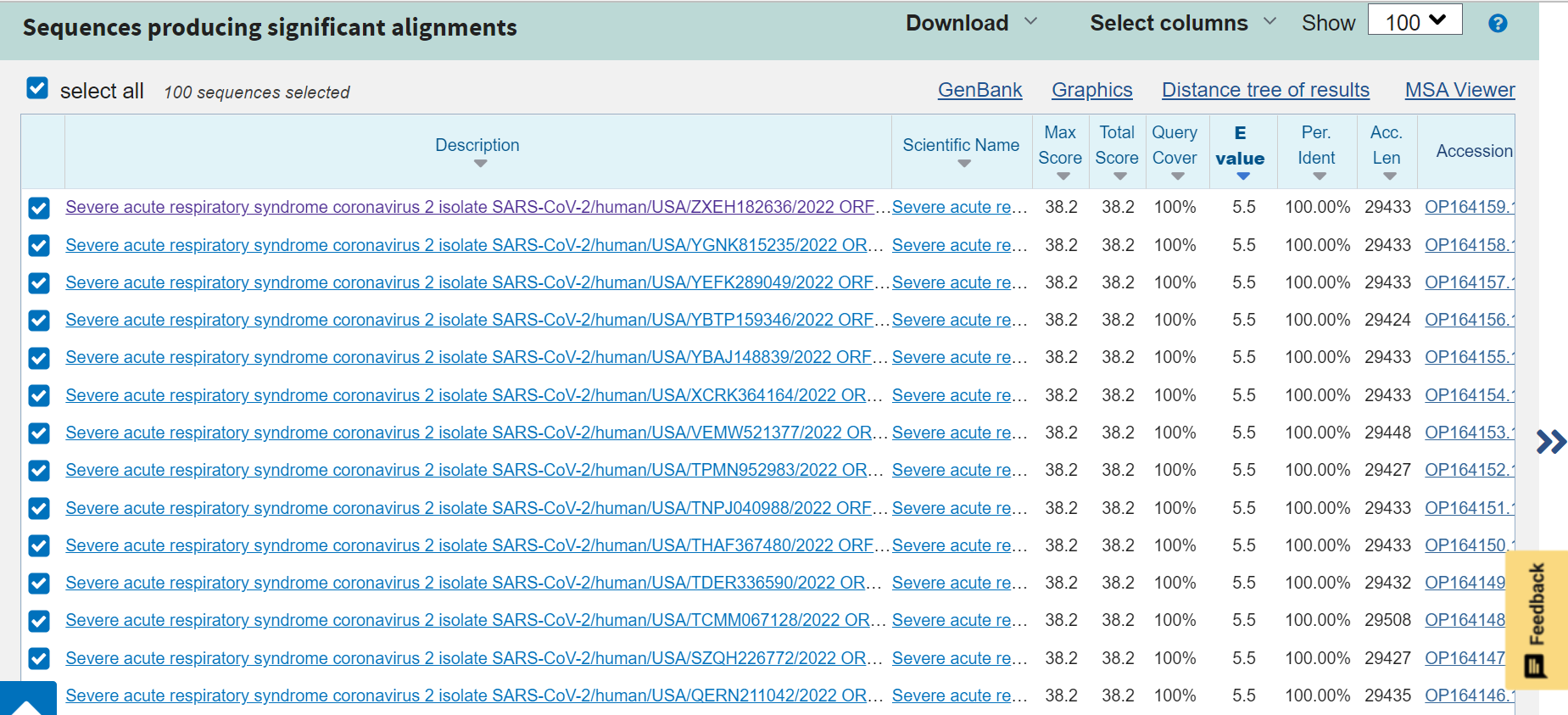

Supplement: Supplemental Information 2 [file peerj-10-14121-s002.zip › Supplemental 1-Blasting results of the LAMP primer sets/Set-6/BIP primer-B2.png]

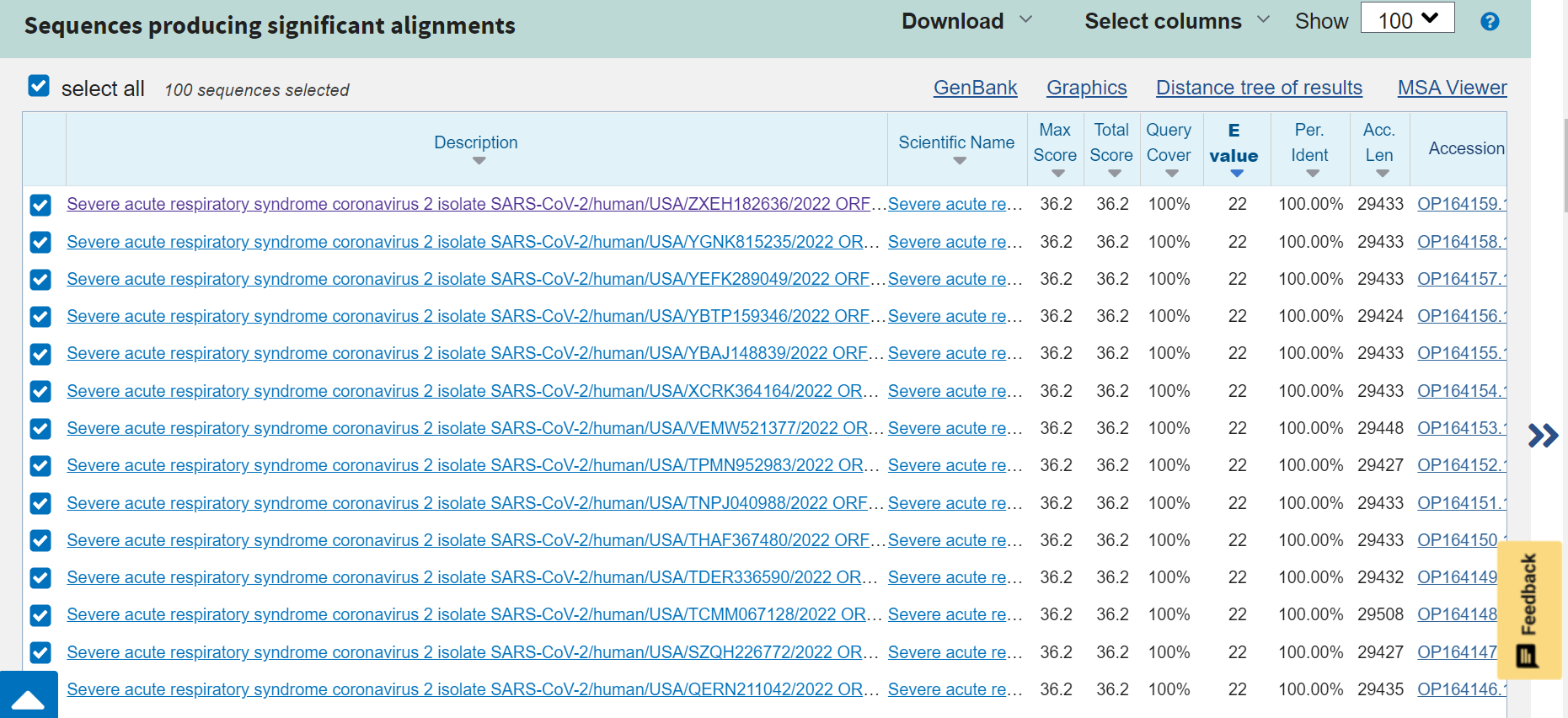

Supplement: Supplemental Information 2 [file peerj-10-14121-s002.zip › Supplemental 1-Blasting results of the LAMP primer sets/Set-6/F3 primer.png]

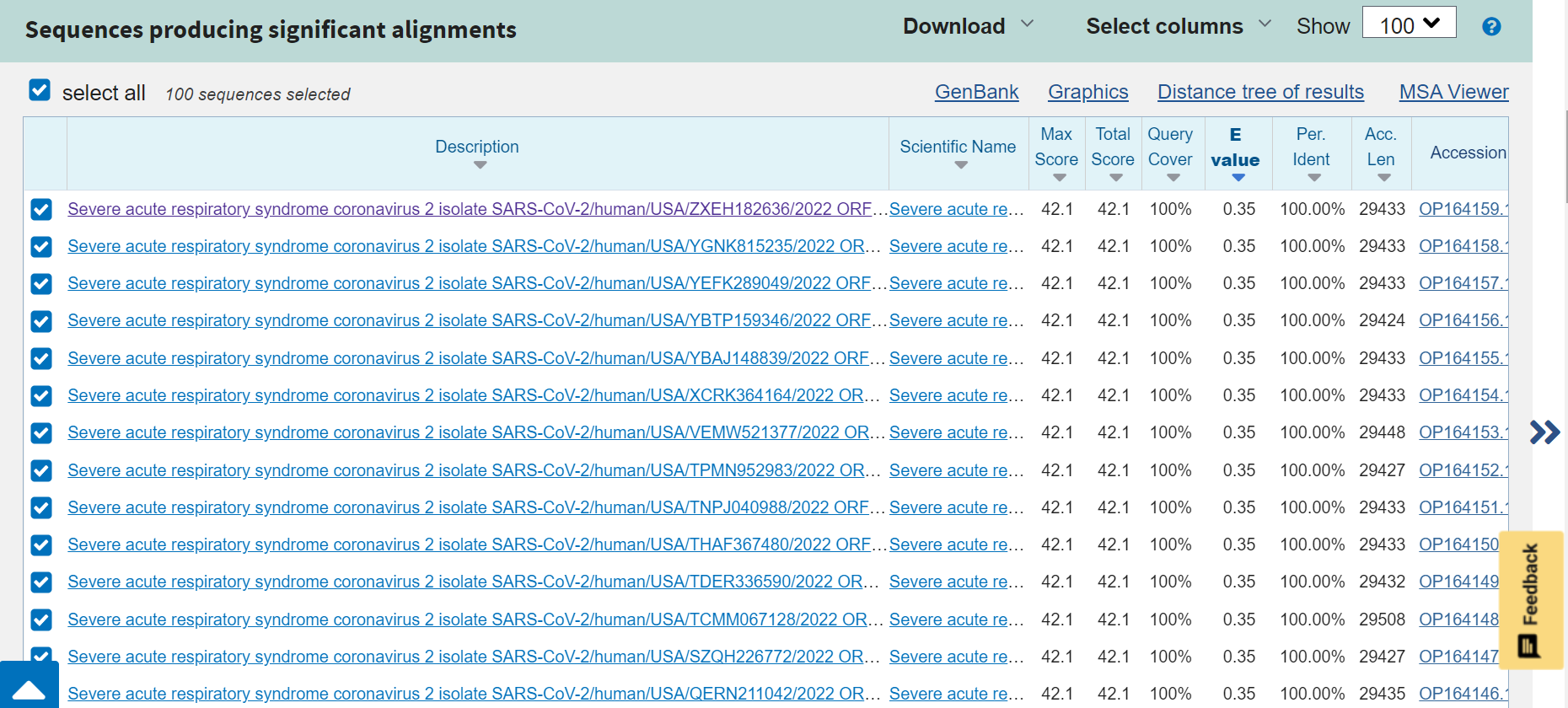

Supplement: Supplemental Information 2 [file peerj-10-14121-s002.zip › Supplemental 1-Blasting results of the LAMP primer sets/Set-6/FIP primer-F1c.png]

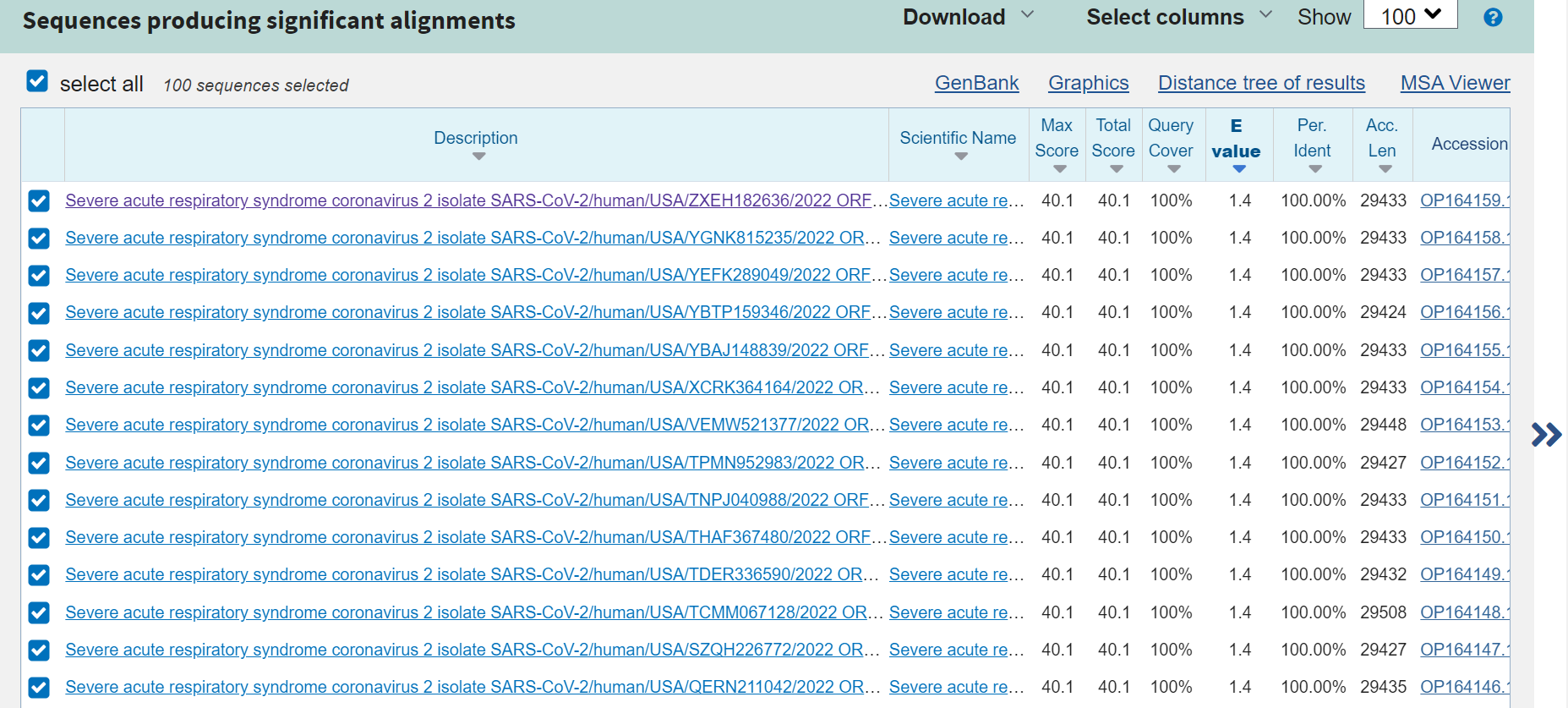

Supplement: Supplemental Information 2 [file peerj-10-14121-s002.zip › Supplemental 1-Blasting results of the LAMP primer sets/Set-6/FIP primer-F2.png]

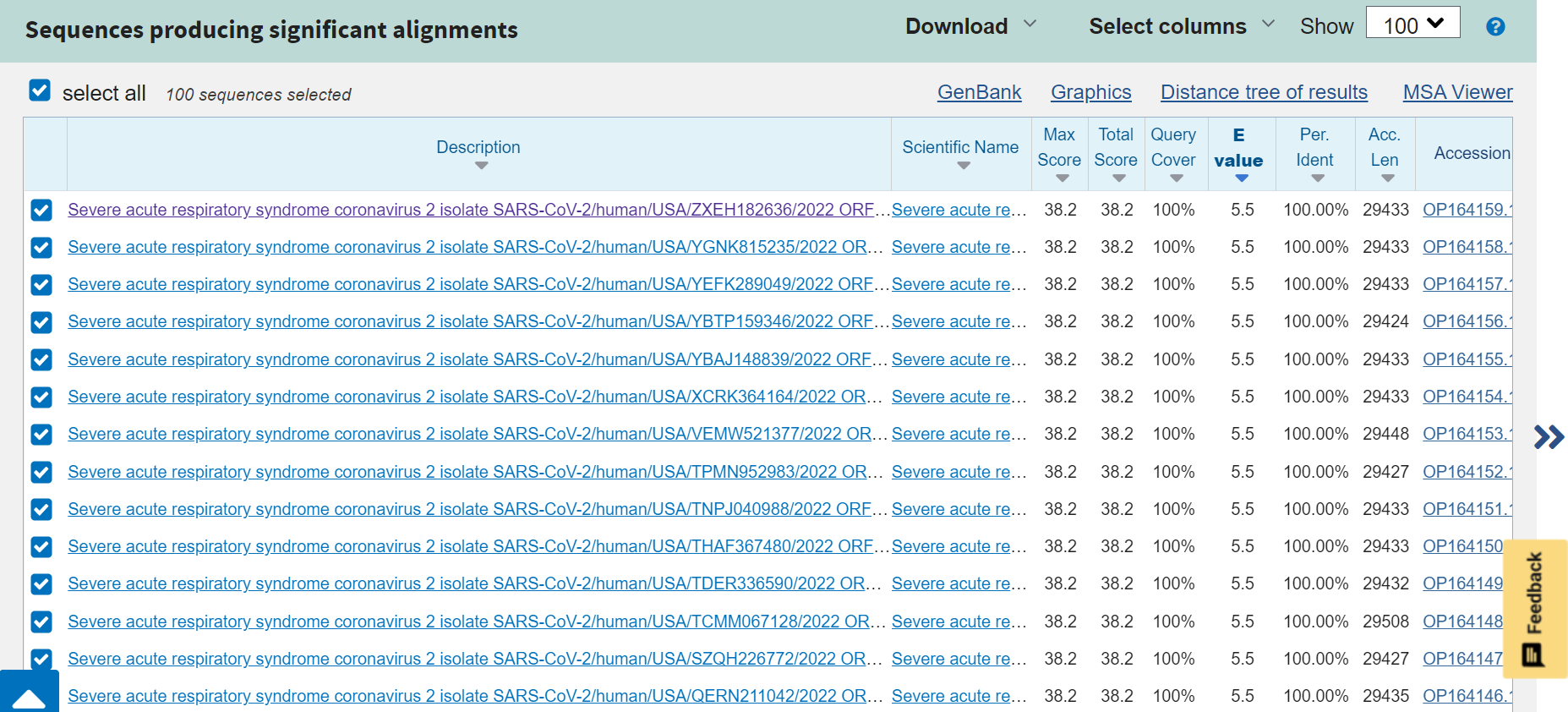

Supplement: Supplemental Information 2 [file peerj-10-14121-s002.zip › Supplemental 1-Blasting results of the LAMP primer sets/Set-6/LB primer.png]

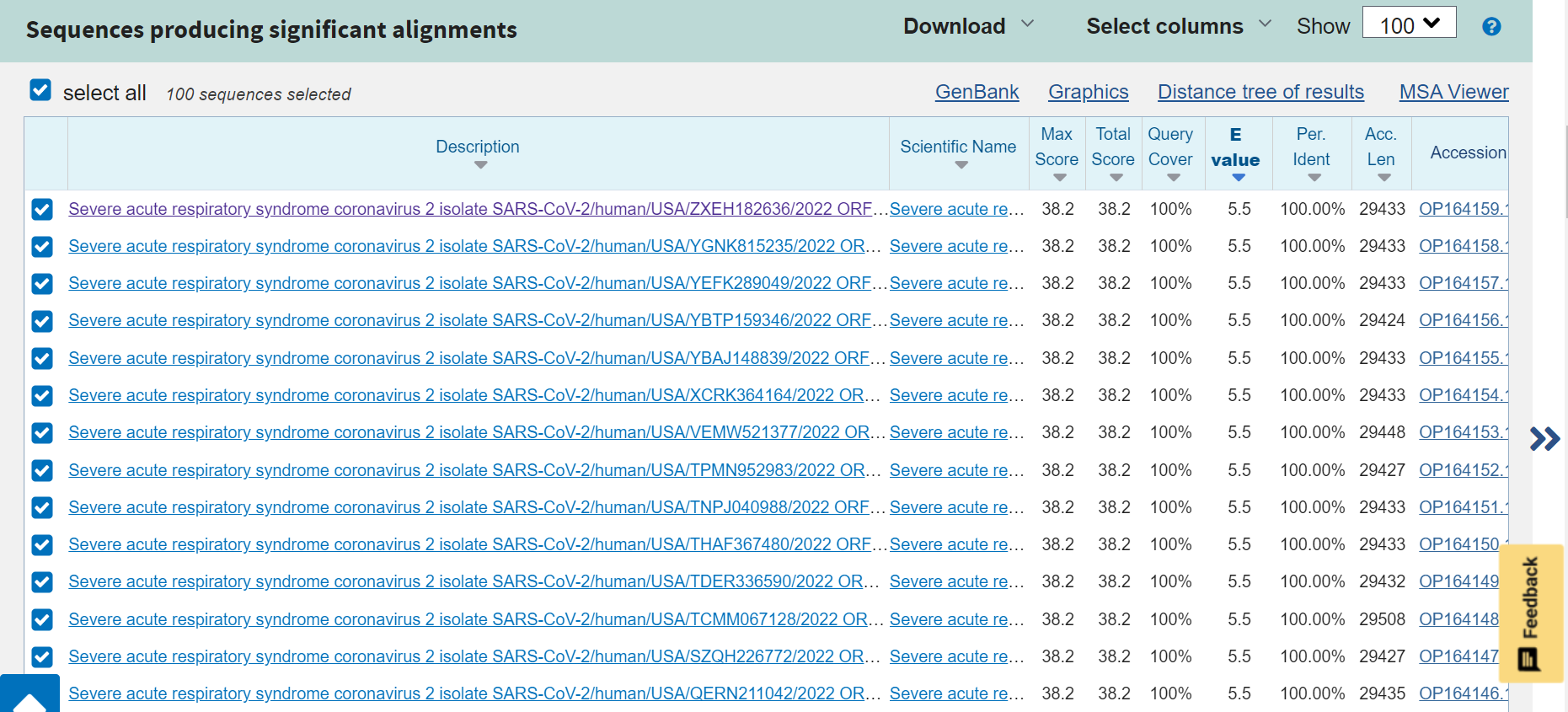

Supplement: Supplemental Information 2 [file peerj-10-14121-s002.zip › Supplemental 1-Blasting results of the LAMP primer sets/Set-6/LF primer.png]

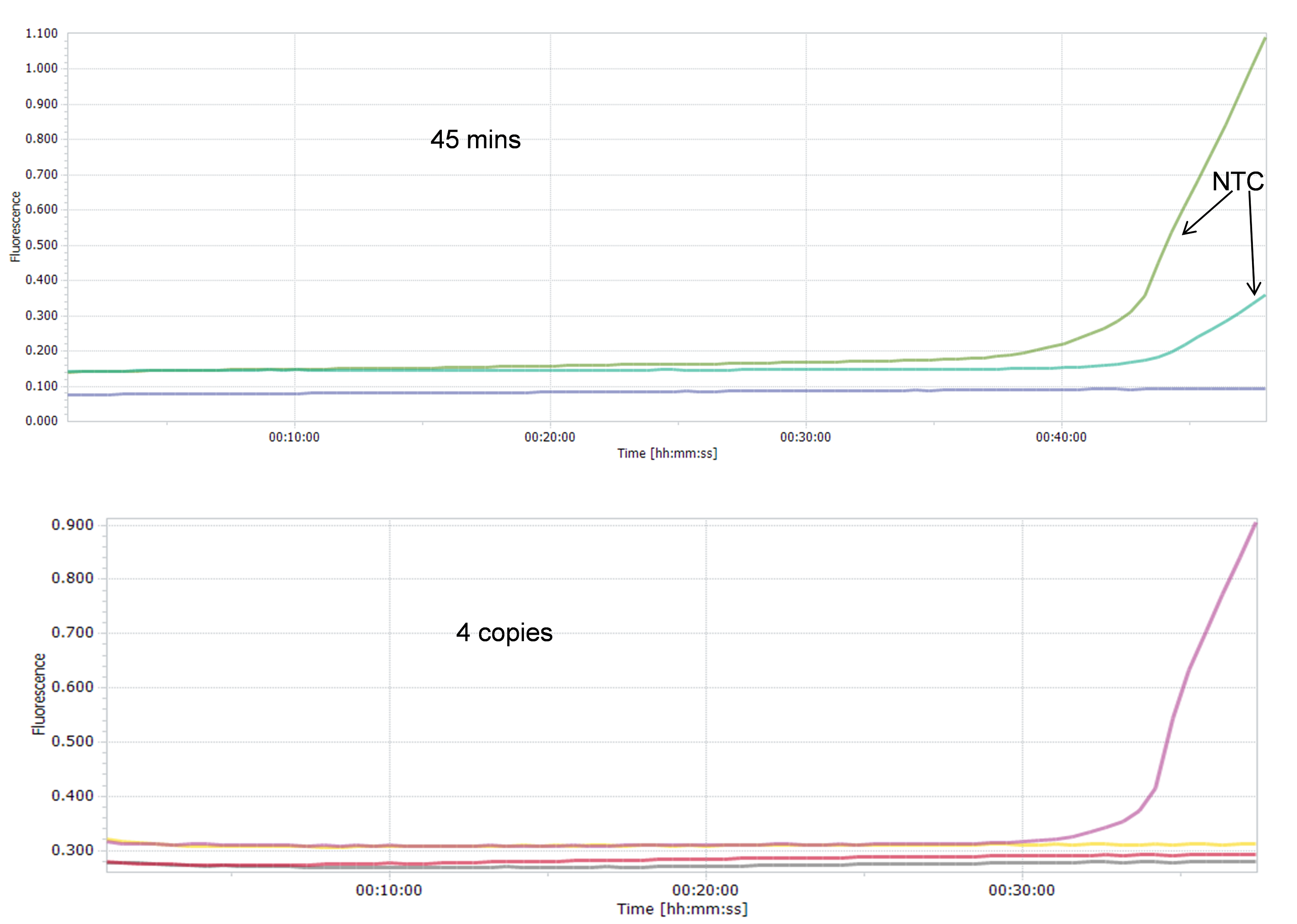

Supplement: Supplemental Information 3 [file peerj-10-14121-s003.png]
